# Effectiveness of Combined Smartwatch and Social Media Intervention on Breast Cancer Survivor Health Outcomes: A 10-Week Pilot Randomized Trial

**DOI:** 10.3390/jcm7060140

**Published:** 2018-06-07

**Authors:** Zachary C. Pope, Nan Zeng, Rui Zhang, Hee Yun Lee, Zan Gao

**Affiliations:** 1 School of Kinesiology, University of Minnesota, 1900 University Ave. SE, Minneapolis, MN 55455, USA; popex157@umn.edu (Z.C.P.); zengx185@umn.edu (N.Z.); zhan1386@umn.edu (R.Z.); 2 College of Pharmacy, and Institute for Health Informatics, University of Minnesota, 8-116 Phillips-Wangensteen Building, 516 Delaware Street SE, Minneapolis, MN 55455, USA; 3 School of Social Work, The University of Alabama, 1022 Little Hall, Box 870314, Tuscaloosa, AL 35487, USA; hlee94@ua.edu

**Keywords:** physical activity, quality of life, social cognitive theory, wearable technology

## Abstract

Physical activity (PA) among breast cancer survivors (BCS) can improve this population’s health and quality of life (QoL). This study evaluated the effectiveness of a combined smartwatch- and social media-based health education intervention on BCS’s health outcomes. Thirty BCS ($\bar{X}$_age_ = 52.6 ± 9.3 years; $\bar{X}$_Wt_ = 80.2 ± 19.6 kg) participated in this 10-week, 2-arm randomized trial, with BCS randomized into: (1) experimental group (*n* = 16): received Polar M400 smartwatches for daily PA tracking and joined a Facebook group wherein Social Cognitive Theory-related PA tips were provided twice weekly; and (2) comparison group (*n* = 14): only joined separate, but content-identical Facebook group. Outcomes included PA, physiological, psychosocial, and QoL variables. Specifically, PA and energy expenditure (EE) was assessed by ActiGraph GT3X+ accelerometers while physiological, psychosocial, and QoL were examined via validated instruments at baseline and post-intervention. No baseline group differences were observed for any variable. Ten BCS dropped out of the study (experimental: 4; comparison: 6). Compared to completers, dropouts differed significantly on several outcomes. Thus, a per-protocol analysis was performed, revealing significant group differences for changes in social support (*t* = −2.1, *p* = 0.05) and barriers (*t* = −2.2, *p* = 0.04). Interestingly, the comparison group demonstrated improvements for both variables while the intervention group demonstrated slightly decreased social support and no change in barriers. Notably, both groups demonstrated similarly increased daily light PA, moderate-to-vigorous PA, EE, and steps of 7.7 min, 5.1 min, 25.1 kcals, and 339 steps, respectively, over time. Despite extensive user training, several experimental BCS found the Polar M400 use difficult—possibly decreasing intervention adherence. Future interventions should utilize simpler smartwatches to promote PA among middle-aged clinical/non-clinical populations.

## 1. Introduction

Invasive or in situ forms of breast cancer were diagnosed among approximately 330,000 women in 2017 [1]. Improved treatment options have led to increased breast cancer survival rates, with 3.1 million breast cancer survivors residing in the U.S. [1,2]. It is noteworthy, however, that studies comparing women never previously diagnosed with breast cancer to breast cancer survivors have observed lower quality of life (e.g., poorer physical functioning; increased depression/anxiety rates; greater fatigue) and poorer physical health among breast cancer survivors [3,4]. While medicinal treatments (e.g., Tamoxifen use) may be prescribed following breast cancer treatment and the beginning of remission, health behavior changes are now more commonly being recommended to breast cancer survivors—the most frequent being increased physical activity participation [3,5,6,7,8,9,10]. Given the ubiquitous nature of modern-day technology, researchers are seeking to leverage several technologies (e.g., smartphone applications, wearable technology, social media) to improve health among various populations through increased physical activity and reduced sedentary behavior [11,12]. Currently, smartwatches are among the most popular technologies being used to assist individuals in living more active and healthier lifestyles.

As a popular form of wearable technology, smartwatches are projected to comprise an approximately $10 billion USD market by 2019 [13]. Smartwatches from companies like Polar, Apple, and Fitbit are most popular within this market due to: (1) attractive price points ($100–250 USD); and (2) the ability to track health metrics like step counts, heart rate, energy expenditure, stairs climbed, and sleep, among other metrics—data which can be sent via Bluetooth to an associated smartphone application for easy interpretation and facilitation of the self-regulation (i.e., tracking and modification) of health behaviors [14,15]. Yet, while some studies [16] have observed smartphone application-based physical activity interventions to be effective in promoting improved physical activity and quality of life among breast cancer survivors, little to no research has been conducted on the effectiveness of smartwatches in the promotion of this population’s health. This is noteworthy as qualitative research has found breast cancer survivors to be interested and open to the use of smartwatches in the self-regulation of physical activity and sedentary behaviors [17].

Of the paucity of high-quality randomized trials which evaluated the effectiveness of smartwatches in the promotion of physical activity, populations investigated have included: overweight and obese individuals [18,19,20], older adults [21], adults [22], and college students [23,24,25]. Findings from these studies have been mixed. For example, Cadmus-Bertram et al. [18,19] observed significantly increased moderate-to-vigorous physical activity and steps/day over 16 weeks among overweight and obese postmenopausal women using the Fitbit to track health behaviors when compared to a control group receiving standard care (e.g., exercise counseling), with marginally positive findings also observed in another study by Thorndike et al. [22] among medical residents and Rote [24] among college students. Yet, other literature among overweight and obese men and women [20], older adults [21], and college students [23,25] has not observed provision of a smartwatch to result in greater improvements in physical activity versus control.

The mixed findings of the preceding studies may be attributable to: (1) little provision of health education despite the need for health literacy in long-term health behavior engagement [26,27]; and (2) lack of an established theoretical framework to guide study design/implementation notwithstanding the importance of health behavior theory in promoting increased intervention effectiveness [28]. This suggests that future randomized trials might be more effective if a theoretically-based health education piece is included. A theory which might be particularly effective in future randomized trials given its concentration on personal-level factors (i.e., self-efficacy, enjoyment, barriers, and outcome expectancy) and micro-environmental factors (i.e., social support) is the Social Cognitive Theory [29,30]. Briefly, this theory posits reciprocal determinism between an individual’s characteristics, environmental factors, and behavior [30]. For example, if an intervention can increase an individual’s self-efficacy for physical activity (i.e., an individual characteristic) and promote greater social support for physical activity (i.e., an environmental factor), this individual is more likely to participate in physically active behavior(s). One manner by which a Social Cognitive Theory-based health education intervention might be delivered is through social media as this technological medium could be used to promote: (1) improvements in individual characteristics via health education; and (2) social support given the inclusion of all intervention participants on intervention-related social media pages upon which the participants can interact and support one another’s health-related endeavors. Data has indicated females to comprise the majority of U.S. Facebook users, with women ≥25 years old representing 40% of all U.S. Facebook users [31]. As the use of theory to frame an intervention’s development/implementation may increase intervention effectiveness [32,33], providing breast cancer survivors with Social Cognitive Theory-based health education tips via Facebook might assist this population in living healthier, more active lifestyles in addition to improving quality of life—a strategy which has, in fact, been suggested by breast cancer survivors in recent research [17].

Therefore, the purpose of this 10-week pilot randomized trial was to evaluate the effectiveness of a combined smartwatch and theoretically-based, social media-delivered health education intervention in promoting improved physical activity participation, physiological/psychosocial health, and quality of life as well as reduced sedentary behavior among breast cancer survivors. By providing experimental group participants a Polar M400 smartwatch and a Social Cognitive Theory-based [29,30], Facebook-delivered health education intervention, it was hypothesized that: (1) experimental group participants would have larger increases in physical activity, energy expenditure, and steps/day in addition to greater decreases in sedentary behavior than comparison group participants receiving only the Facebook-delivered health education intervention given the experimental group’s additional ability to monitor Polar M400 health metrics like steps per day and daily activity duration; (2) experimental group participants would experience greater improvements in weight, body composition, and cardiorespiratory fitness versus comparison. This was hypothesized as it was believed the experimental group might modify caloric consumption and increase physical activity participation based upon the Polar M400’s energy expenditure and steps per day/daily activity time readings, respectively—behaviors which would be supplemented by the health education being delivered via this group’s respective Facebook group; and (3) more favorable changes in Social Cognitive Theory-related psychosocial constructs and quality of life would be observed in the experimental group versus comparison partially resulting from the greater hypothesized changes for physical activity and physiological outcomes among the experimental group. Observations may assist health professionals in developing large-scale, low-burden, and well-integrated physical activity interventions among breast cancer survivors and other clinical populations which can effectively improve health outcomes during or following treatment.

## 2. Materials and Methods

This manuscript’s construction was guided by the CONSORT guidelines [34] for the reporting of randomized trials.

### 2.1. Study Design

A 10-week two-arm parallel randomized pilot trial study design was implemented, with data collected from November 2016 to April 2017. Baseline and 10-week assessments of 7-day habitual physical activity and sedentary behavior as well as evaluations of physiological, psychosocial, and quality of life outcomes were performed. Given the pilot nature of the trial, use/acceptability of the intervention was also assessed. Notably, the current investigation built upon the researchers’ previous smartphone- and social media-based health education intervention study [16], with three distinct differences. First, the current study used the Polar M400 smartwatch as opposed to a smartphone application because most breast cancer survivors in the previous study stated the need to open their smartphone to track/document physical activity was burdensome and that the “always on” physical activity tracking capabilities of smartwatches would be preferable during future interventions. Second, the Facebook-delivered health education intervention used in the current study, while similar to the previous study, included the addition of a workout program (see Procedures) designed around the unique limitations of breast cancer survivors (e.g., functional limitations imposed by mastectomies and/or comorbidities). Finally, the previous study was a single group pre-post intervention design, not a randomized trial as implemented in the present investigation. All procedures performed with participants were in accordance with the standards of the Institution and/or national research committee and with the 1964 Helsinki declaration and its later amendments or comparable ethical standards [35]. Testing was not performed until University institutional review board approval and participant informed consent were obtained.

### 2.2. Recruitment and Inclusion/Exclusion Criteria

Posted flyers in the University’s Masonic Cancer Center and surrounding medical buildings, University-wide emails, online postings, and word-of-mouth were all used to recruit eligible breast cancer survivors. Breast cancer survivors interested in study participation contacted one of the researchers (ZCP) and were screened against the following criteria: (1) females of any race/ethnicity; (2) ≥21-years-old; (3) prior stage 0–III breast cancer diagnosis; (4) breast cancer treatment finished 3 months to 10 years earlier with no recurrence; (5) possessed an active Facebook account; and (6) willingness to complete the Physical Activity Readiness Questionnaire [36] and be randomized into an experimental or comparison group. Exclusion criteria were: (1) any ongoing breast cancer treatment; and (2) contraindication(s) to physical activity participation (e.g., pacemaker implant, medical condition) as indicated by the Physical Activity Readiness Questionnaire which could potentially limit study participation.

### 2.3. Measures

Demographic/clinical variables. Breast cancer survivors self-reported age, race/ethnicity, birthplace, education/annual income level, marital/employment status, breast cancer diagnosis stage, treatment type, months since diagnosis, remission duration, and Tamoxifen use.

#### 2.3.1. Primary Outcome

Physical activity levels/energy expenditure. ActiGraph GT3X+ accelerometers were employed at baseline and 10 weeks to evaluate: mean daily duration of sedentary behavior, light physical activity, and moderate-to-vigorous physical activity as well as energy expenditure in kcalories and steps/day. Previous research [37] has observed the ActiGraph GT3X+ to be valid in physical activity measurement among adults in free-living conditions. Per recommendations made in previous literature [38], breast cancer survivors wore the accelerometer for 7 days to ensure collection of physical activity data on at least 2 weekdays and 1 weekend day. Data was analyzed using the following empirically-based cut points in counts/minute: sedentary behavior: 0–99; light physical activity: 100–2019; moderate-to-vigorous physical activity: ≥2020 [39]. Any day with less than 10 h of valid wear time for any participant was excluded from the analysis [38].

#### 2.3.2. Secondary Outcomes

Anthropometry, body composition, and cardiorespiratory fitness. To measure height to the nearest half-centimeter and weight/body fat percentage, trained research assistants used a Seca stadiometer (Seca, Hamburg, Germany) and a Tanita BC-558 IRONMAN^®^ Segmental Body Composition Monitor (Tanita, Tokyo, Japan), respectively. Validity of bioelectrical impedance for field measurements of body fat percentage has been observed in other adult populations [40]. Finally, the YMCA 3-min Step Test was used to evaluate cardiorespiratory fitness, with palpation of the radial artery for 1 min following the test to acquire a post-test heart rate in beats/minute [41]. These measurements were taken at baseline and 10 weeks.

Psychosocial variables. Psychometrically validated questionnaires were used to assess social support, barriers, self-efficacy, enjoyment, and outcome expectancy. In detail, a 5-item social support measure adapted from the Patient-Centered Assessment and Counseling for Exercise questionnaire [42] queried breast cancer survivors regarding how often significant others encouraged them to be physically active using a 5-point Likert-type scale (1: almost never to 5: almost always). For physical activity barriers, breast cancer survivors rated the degree of agreement between personal barriers and hypothetical barriers on a 14-item measure which employed a 4-point Likert-type scale (1: strongly disagree to 4: strongly agree) [43]. A 9-item measure developed by Rodgers et al. [44] examined breast cancer survivors’ self-efficacy as they rated how confident they felt in specific exercise situations (e.g., “… exercise when you feel discomfort” or “… exercise when you lack energy”) using a percentage scale (0%: not confident at all to 100%: extremely confident in 10% increments). A modified 5-item measure constructed by Harter [45] evaluated physical activity enjoyment as breast cancer survivors rated their agreement with statements like “Engaging in physical activity is the thing I like to do best” using a 5-point Likert-type scale (1: strongly disagree to 5: strongly agree). Finally, a 9-item measure developed by Trost et al. [46] assessed breast cancer survivors’ outcome expectancy as they rated agreement with responses originating from the stem “If I was to exercise on most days it would …”, with sample responses like “give me more energy” and “help to control my weight”. This questionnaire employed a 5-point Likert-type scale (1: strongly disagree to 5: strongly agree). These questionnaires were administered at baseline and 10 weeks.

Quality of life. Evaluation of physical functioning, anxiety, depression, fatigue, sleep, ability to participate in social roles/activities, and pain occurred via the Patient Reported Outcome Measurement Information System [47], with all outcomes assessed via 5-point Likert-type scales aside from that of pain intensity. Specifically, to assess physical functioning, breast cancer survivors rated how current physical abilities (e.g., “Are you able to get in and out of bed?”) were made more difficult due to current health (1: without any difficulty to 5: unable to do). A 7-day recall of symptom frequency (1: never to 5: always) was used to assess anxiety, depression, and ability to participate in social roles/activities. Symptom frequency was also reported for fatigue, sleep, and pain, with responses ranging from 1: not at all to 5: very much. Finally, sleep quality was evaluated using a 5-point Likert-type scale (1: very poor to 5: very good), with pain intensity assessed on a 0 to 10 scale (0: no pain to 10: worst pain imaginable). Prior research has indicated the validity of the Patient Reported Outcome Measurement Information System in clinical populations [48]—including cancer populations [49]. This questionnaire was administered at baseline and 10 weeks.

Use/acceptability. A post-intervention self-reported survey among experimental participants evaluated: weekly frequency of Polar M400 wear, weekly frequency/mean duration of Polar M400 use during exercise, and Polar M400 enjoyment (dichotomous “yes” or “no” response). Experimental participants were also asked to list any negative features of the smartwatch. Moreover, both groups were surveyed at post-intervention regarding: implementation frequency of Facebook-delivered health education tips and whether they perceived the health education tips as helpful (dichotomous “yes” or “no” response).

### 2.4. Procedures

Breast cancer survivors interested in participating contacted a study researcher (ZCP), with potential participants screened against inclusion criteria. Baseline testing was then scheduled for eligible breast cancer survivors. Baseline testing began with a battery of questionnaires evaluating demographic/clinical characteristics, psychosocial constructs, and quality of life indices. Next, breast cancers survivors’ height, weight, body composition, and cardiorespiratory fitness were measured. Participants were then given an ActiGraph GT3X with instructions on how and when the accelerometer needed to be worn over the following 7 days. During the 7 days the breast cancer survivors wore the accelerometer during baseline testing, a random numbers table was used to randomize participants into the experimental or comparison group, with a 1:1 allocation ratio. Upon returning the accelerometer, each participant met with the researcher (ZCP) to learn their group allocation and discuss use of the Polar M400 and/or the Facebook group components of their respective intervention.

Experimental group participants were instructed first on the use of the Polar M400—a powerful no-frills smartwatch capable of tracking health metrics such as energy expenditure, steps/days, and daily physical activity duration, among other metrics. Notably, while the Polar M400 is equipped with a triaxial accelerometer, the smartwatch also possesses global positioning system capabilities and Bluetooth compatibility—the latter allowing the smartwatch to sync to an associated smartphone application and/or internet-based portal [50]. Given the Polar M400’s numerous functions, the researcher spent approximately 15 min with each experimental participant providing a tutorial of the smartwatch’s functions, subsequently providing the Polar M400 manual to experimental group participants as well. Next, experimental group participants were given a tutorial of the Facebook page used throughout the intervention to provide twice-weekly Social Cognitive Theory-related health education tips (see Supplementary Materials). These tips were developed to assist participants’ integration of physical activity into their daily routine by improving participants’ physical activity-related self-efficacy, outcome expectancy, social support, and enjoyment while reducing barriers. These tips have been used with success in a previous intervention among breast cancer survivors [16]. For example, health education tips written to increase participants’ outcome expectancy, enjoyment, and social support used empirically-based facts to remind participants of the improved mood/quality of life and physiological outcomes which may occur due to increased physical activity participation while also providing some ideas by which to make physical activity more fun and social. Experimental group participants were also told that they could post physical activity-related statistics to the Facebook group from their Polar M400 and/or comment within the Facebook group at their discretion to support one another toward physical activity goals.

Comparison group participants received identical instructions to those listed above regarding accessing and using a separate, content-identical Facebook group, with each participant asked to discontinue smartwatch use throughout the duration of the study. Finally, both groups also received a periodized strength and aerobic training program (see Supplementary Materials) via the Facebook group developed by the first author (an ACSM Certified Exercise Physiologist) with the physical limitations of breast cancer survivors—particularly those of the upper body—accounted for. This workout program was not mandatory to implement, however. Notably, both Facebook groups were completely private (i.e., closed) and unsearchable via Facebook. To ensure intervention fidelity, both groups were contacted every other week throughout the study and encouraged to continue using the Polar M400 (experimental group) and/or reading and attempting to implement the Facebook-delivered health education tips (both groups). Successful study completion resulted in receipt of a $100 gift card compensation. Experimental group participants were not allowed to keep the Polar M400 following study completion. 

### 2.5. Statistical Analysis

First, intervention use/acceptability was evaluated descriptively—providing context for subsequent results. Second, descriptive statistics for all other outcomes at each time point were calculated, with an outlier analysis and Shapiro–Wilks tests of normality also performed. To evaluate baseline group differences in each categorical and continuous variable, chi-square and independent *t*-tests were then conducted, respectively. Third, mean change for each outcome variable was calculated by subtracting the value measured at baseline from the measurements taken at 10 weeks. Finally, Mann–Whitney U tests and independent *t*-tests were used to investigate group differences over time in the mean change of primary (i.e., moderate-to-vigorous physical activity, light physical activity, steps/day, and energy expenditure) and secondary (i.e., weight, body fat percentage, cardiorespiratory fitness, psychosocial constructs, and quality of life) outcomes. Notably, non-normal data distributions were observed for all physical activity outcomes and quality of life outcomes and, therefore, Mann–Whitney U tests were employed to evaluate differences in mean change between groups over time. Normally distributed data distributions were observed for physiological and psychosocial variables; thus, independent *t*-tests assessed differences in mean change over time in these outcomes, with Levene’s Test for Equality of Variance used to examine homogeneity and determine the correct *p*-value(s) to report. Given the exploratory nature of the pilot trial, alpha was not adjusted, remaining at a *p*-value of 0.05 for all analyses.

## 3. Results

### 3.1. Baseline Comparisons and Participant Flow

**Figure 1:**
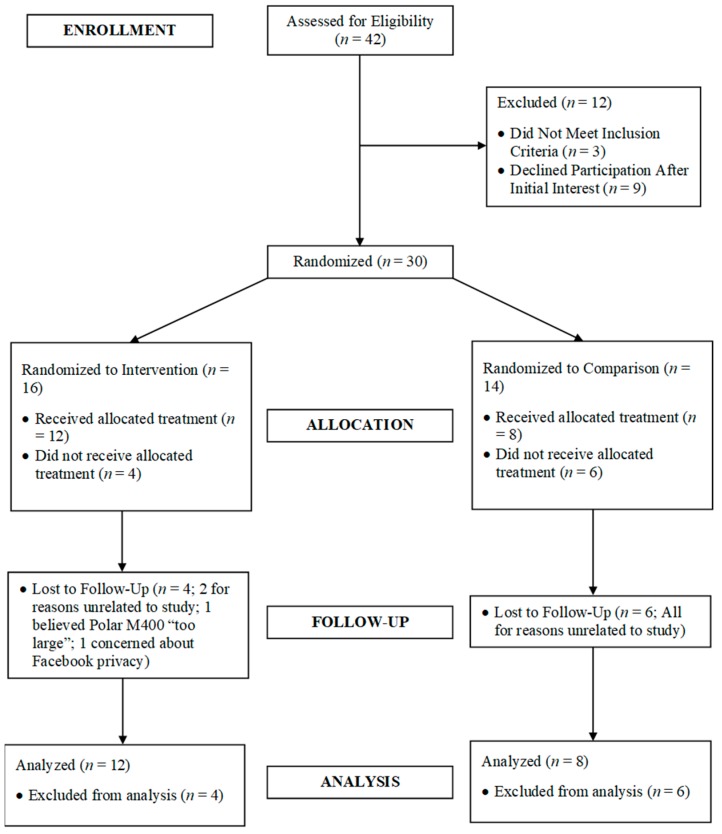
CONSORT study participant flow diagram.

Participant flow through the study is outlined within the CONSORT Diagram (see Figure 1). Forty-two breast cancer survivors expressed interest in the study, with 30 breast cancer survivors subsequently found eligible for baseline testing and randomization. Baseline values for these breast cancer survivors’ demographic and clinical outcomes are presented in Table 1, with baseline comparisons of physical activity, physiological, psychosocial, and quality of life outcomes included in Table 2. No baseline group differences were observed for any variable suggesting efficacy of the randomization procedures.

Ten breast cancer survivors dropped out of the study (66.6% retention rate; experimental group: 4; comparison group: 6). Eight breast cancer survivors dropped out due to reasons unrelated to the study. Regarding the other two dropouts, one participant dropped out due to the perception that the Polar M400 was “too large” and inhibiting her daily work, with the other dropping out due to concerns about Facebook privacy (despite the Facebook groups being entirely private and unsearchable). Compared to completers, dropouts had less private insurance coverage, longer duration since diagnosis, and lower annual income, daily light physical activity, moderate-to-vigorous physical activity, EE, and steps. Given these differences, a per-protocol analysis was completed when evaluating changes in health outcomes from baseline to 10 weeks.

### 3.2. Intervention Use/Acceptability

All experimental group participants reported wearing the Polar M400 6–7 days/week. The weekly mean frequency and duration per session for the use of this device during exercise was 4.55 ± 1.74 sessions/week and 53.9 ± 16.7 min/session, respectively. Only 7 of the 12 experimental participants reported enjoying Polar M400 use, however, with these participants stating the following as negative device features: was difficult to sync to phone/computer; had trouble tracking activities like biking and swimming; the size of the smartwatch was “too big”, with most breast cancer survivors stating the device’s buttons would get inadvertently pressed when dressing and undressing given the thickness of the smartwatch; and the smartwatch use was not as “straightforward” to use as other smartwatches as the device used buttons on the side of the device to toggle through black-and-white screens and did not possess a color touchscreen with easily accessible tabs akin to other smartwatches like the Fitbit. Regarding the Facebook health education intervention, participants across both groups reported implementing the tips provided 1.2 ± 1.0 times/weekly, with 16 out of 20 participants enjoying these health education tips.

#### 3.2.1. Primary Outcomes

Table 3 contains descriptive statistics for breast cancer survivors’ mean daily moderate-to-vigorous physical activity, light physical activity, sedentary behavior, energy expenditure, and steps/day at baseline and 10 weeks. No statistically significant group differences were observed for change over time for any variable. However, both the experimental and comparison groups demonstrated increased mean daily moderate-to-vigorous physical activity (+3.5 and +7.5 min, respectively), light physical activity (+7.5 and +8.1 min, respectively), energy expenditure (+26.7 and +22.6 calories, respectively), and steps/day (+342.7 and +334.4 steps/day, respectively) from baseline to 10 weeks. Notably, mean daily sedentary behavior remained stable over time, but slight increases were observed within the experimental (+2.4 min) and comparison (+0.4 min) groups.

#### 3.2.2. Secondary Outcomes

Table 4 provides descriptive statistics for all physiological and psychosocial outcomes at baseline and 10 weeks while Table 5 provides descriptive statistics for all quality of life outcomes at the same time points.

### 3.3. Physiological Changes over Time

Breast cancer survivors’ weight was observed to be largely unchanged within both groups from baseline to 10 weeks, but the comparison group did demonstrate a larger, albeit non-significant (*p* > 0.05), decrease in body fat percentage (−1.0%) compared to the experimental group (+0.5%) during the intervention. Additionally, while improvements in cardiorespiratory fitness over time were not significantly different between groups, both groups demonstrated reduced heart rate following the YMCA 3-min Step Test at 10 weeks (experimental: −4.8 beats/minute; comparison: −4.5 beats/minute).

### 3.4. Psychosocial Construct Changes over Time

Significant group differences for changes in social support (*t* = −2.1, *p* = 0.05) and barriers (*t* = −2.2, *p* = 0.04) were observed. Interestingly, from baseline to 10 weeks, the comparison group demonstrated improved physical activity-related social support and decreased barriers while the experimental group demonstrated slightly decreased social support and no change in barriers.

### 3.5. Quality of Life Changes over Time

No significant group differences over time were observed for any quality of life outcome. It is noteworthy, however, that the experimental group demonstrated decreased social role limitations and reductions in sleep disturbances, with a subsequent increase in sleep quality, from baseline to 10 weeks.

## 4. Discussion

Breast cancer survivors are at risk of greater physical inactivity, poorer physiological/psychological health, and reduced quality of life due to past breast cancer treatment [3,4]. Given physical activity’s demonstrated effects on breast cancers survivors’ health outcomes, health behavior change interventions have become common recommendations among this population [5,6,7,8,9,10]. As breast cancer survivors have found acceptable and expressed interest in the use of smartwatches to self-regulate physical activity and sedentary behavior [17], this pilot randomized trial examined the effectiveness of a combined smartwatch- and social media-based health education intervention in the promotion of improved physical activity and health indices among breast cancer survivors. Observations suggested this type of intervention might promote improvements in physical activity and certain health outcomes, but that smartwatch complexity needs to be considered as the experimental group did not demonstrate significantly different improvements over time versus the comparison group.

The current investigation’s first hypothesis was that greater increased physical activity, energy expenditure, and steps/day in addition to reduced sedentary behavior would be observed among the experimental group versus the comparison group. Observations were not congruent with this hypothesis as both groups demonstrated similarly increased moderate-to-vigorous physical activity, light physical activity, energy expenditure, and steps/day during the intervention period with mean increases of approximately 5 min, 8 min, 25 kcalories, and 340 steps/day, respectively. Despite no statistically significant group differences, these findings may have some clinical/practical significance. Specifically, research has indicated sedentary behavior to increase breast cancer recurrence risk by up to 34% among breast cancer survivors [51]. Reasons for the increased recurrence risk are many, not the least of which has to do with adiposity and its effects on cancer-related hormones. Briefly, increased adiposity among breast cancer survivors has been positively correlated with insulin resistance as well as poorer regulation of insulin growth factor-1 (IGF-1) and estrogen—two hormones frequently implicated in breast cancer development and recurrence [52]. Fortunately, physical activity has been shown to promote appropriate regulation of insulin, IGF-1, and estrogen [53]. Thus, the combined ~13 min/day increase in moderate-to-vigorous physical activity and light physical activity from baseline to 10 weeks is noteworthy as this increase contributed to ~1.5 h/week more of physical activity among the current sample. Nonetheless, it is necessary to question why the experimental group did not experience greater increases in the preceding primary outcomes than the comparison group.

Examination of the experimental groups’ opinion of the Polar M400 smartwatch may provide some answers. Explicitly, five out of the 12 breast cancer survivors in the experimental group expressed frustration with the smartwatch, with participants finding the smartwatch too complex, hard to connect to the associated smartphone application, and too big. These remarks are congruent with recent literature among breast cancer survivors during which it was found breast cancer survivors prefer simple, easy-to-use smartwatches with larger screens when using these devices to self-regulate physical activity and sedentary behavior [17]. Indeed, while powerful, the Polar M400 is not as intuitive as other smartwatches available on the market (e.g., Apple Watch 3, Fitbit Ionic) given the fact that Polar has long marketed their products to the sport performance industry and has only recently begun to develop and market smartwatches. Therefore, future studies among breast cancer survivors may utilize smartwatches which are more mainstream and easier-to-use—keeping in mind that breast cancer survivors are often middle-aged females and that research does suggest a negative relationship between age and technological literacy, adoption, and use [54,55].

The preceding observations regarding the usability of the Polar M400 might have also influenced the conclusions made regarding the study’s second hypothesis wherein it was stated that the experimental participants would have greater improvements in physiological outcomes versus comparison participants. Indeed, no difference in mean body weight changes were observed between groups, with the lower heart rate observed following the step test nearly identical for both groups (approximately 5 beats/minute; indicative of improved cardiorespiratory fitness). Interestingly, decreased body fat percentage was observed among comparison participants while slightly increased body fat percentage was seen among experimental participants during the intervention. Yet, it is difficult to know whether these changes represent actual changes in body fat percentage due to the intervention as the magnitude of these changes is within the margin of error commonly reported for bioelectrical impedance testing on individuals of higher body fat percentage akin to that observed in the current investigation [56]. While the lower heart rate following step testing is promising given the fact improved cardiorespiratory fitness has been observed to negatively correlate with breast cancer survivors’ risk of breast cancer recurrence [57], decreased body weight and body fat percentage is still highly desired given the aforementioned influence of adiposity on key cancer hormones [52,53].

The final hypothesis of this study was that the experimental participants would experience greater beneficial changes in social cognitive theory-related psychosocial constructs and quality of life indices versus the comparison group. Observations, again, were not congruent with this hypothesis. Interestingly, significantly greater increases in social support were observed among comparison participants versus experimental participants who demonstrated slightly decreased social support over time. It is noteworthy, however, that experimental participants had marginally significantly higher social support at baseline versus comparison participants (*p* = 0.06), with both groups completing the intervention with approximately the same social support scores. Therefore, these paradoxical findings might be attributed more to regression towards the mean than the effects of the intervention. Notably, experimental participants did demonstrate greater reductions in social role limitations and sleep disturbances in addition to a slight increase in sleep quality versus comparison participants during the intervention. These observations are positive as social role limitations and fatigue (among other factors) have been observed to be a major determinants of quality of life among breast cancer survivors [58]. Moreover, sleep disturbances have been shown to moderate the effect of vasomotor symptoms associated with the onset of breast cancer treatment-induced menopause (e.g., hot flashes) on depressive symptoms among breast cancer survivors approximately the same age as women in the current study [59]. This suggests the less frequent sleep disturbances (and improved sleep quality) indicated among experimental participants might confer health benefits in the long term. Nonetheless, more research with larger and more diverse samples is needed to investigate how to effectively promote improved psychosocial and quality of life health outcomes among breast cancer survivors.

Strengths of the current study include: (1) investigation of breast cancer survivors—a population not often targeted for physical activity interventions; (2) use of a combined smartwatch and social media-delivered health education intervention; (3) utilization of Social Cognitive Theory to develop and implement the intervention; and (4) evaluations of physiological, psychosocial, and quality of life indices in addition to a full spectrum of objectively-assessed physical activity analyses (i.e., durations of sedentary behavior, light physical activity, and moderate-to-vigorous physical activity). Despite these strengths, limitations are present and should be accounted for when interpreting the study’s findings. To begin, the study was not blinded. Although group allocation was concealed as best as possible from participants, several breast cancer survivors in the present study knew one another from various breast cancer survivors support groups raising the concern of contamination between groups. Second, while the request was made to comparison participants to discontinue smartwatch use throughout the duration of the intervention, it might be possible that some participants did not follow these directives or utilized other means of health behavior tracking in place of a smartwatch (e.g., a smartphone-based health application). Third, the study concentrated exclusively on promoting physical activity and reducing sedentary behavior, with no dietary component included. Diet has been cited as an important consideration when seeking to promote improved health among breast cancer survivors given the dietary effects which can be present on hormones related to breast cancer development/metastasis such as estrogen, insulin, and IGF-1 [53]. As interventions targeting both dietary and physical activity behaviors have been shown to be more effective than interventions targeting dietary or physical activity behaviors exclusively [60], including both emphases in future interventions is advised. Further, measurements of biomarkers in future studies might also be advised to increase the generalizability of the effects an intervention of this type might have in a clinical setting. Indeed, not only have biomarkers like estrogen, insulin, and IGF-1 been implicated in breast cancer development/recurrence [52,53] but, more recently, myokines have been observed important to breast cancer development [61]. Specifically, this latter research has indicated that muscular contraction can increase myokine secretion which may have a therapeutic effect on organ metabolism and potentially reduce the likelihood of breast cancer recurrence among breast cancer survivors. Myokine measurements might also be correlated with lean mass measurements made by body composition assessment techniques with demonstrated high accuracy like hydrodensiometry and dual x-ray absorptiometry [62]. Nonetheless, the current study’s methodology was sufficient to investigate whether a combined smartwatch- and theoretically-based, social media-delivered health education intervention was feasible among breast cancer survivors. Although ActiGraph GT3X+ accelerometer has demonstrated acceptable validity and reliability in assessing sedentary and physical activity behavior among adults, more accurate motion sensor in measuring sedentary and light physical activity (e.g., activPAL) may be used in future studies with this population [63]. Additionally, the small sample size limited statistical power, with the fact the sample was well-educated and of high socioeconomic status limiting study generalizability. Larger and more diverse samples are suggested for future investigations. Finally, given the increasing popularity of the Social Ecological Model and the important role the neighborhood environment plays in enhancing physical activity and quality of life, researchers might include neighborhood environment in the research design in the future [64].

## 5. Conclusions

The current study suggests that a theoretically based health education intervention delivered using social media may be able to promote increased physical activity and select improved health indices among breast cancer survivors. However, observations do not suggest smartwatch use confers any additional benefit to an intervention of this type. Indeed, despite extensive user training, most experimental participants found the Polar M400 difficult to use—possibly decreasing intervention adherence. Future interventions should utilize simpler smartwatches to promote PA among middle-aged clinical/non-clinical populations as technology-based interventions of this type still show promise in providing low-burden, well-integrated health promotion options for these populations.

## Figures and Tables

**Table 1:** Participant baseline comparisons for clinical and demographic characteristics *.

Demographic Characteristics (*n* = 30)					
	Experimental (*n* = 16)		Comparison (*n* = 14)		
	Avg. (*M* ± SD)	Freq. (Counts)	Avg. (*M* ± SD)	Freq. (Counts)	*p*-Value
Age (years)	50.6 ± 7.4		54.9 ± 11.0		0.23
Race/ethnicity					0.28
● Caucasian	16		13		
● Hispanic	0		1		
Educational status					0.98
● Some college/technical school		2		2	
● College graduate		5		4	
● Graduate school		9		8	
Health insurance					0.12
● Private		16		12	
● Medicaid		0		2	
Employment status					0.46
● Full time		9		7	
● Part time		5		4	
● Retired		0		2	
● Housewife		2		1	
Marital status					0.60
● Married		14		10	
● Separated/divorced		1		2	
● Widowed		0		1	
● Living with unmarried partner		1		1	
Annual income (USD)					0.65
● $10,001–20,000		1		1	
● $30,001–40,000		1		2	
● $40,001–50,000		0		1	
● $50,000–74,999		0		1	
● $75,000–99,999		4		3	
● ≥$100,000		10		6	
**Clinical Characteristics (*n* = 30)**					
Time in remission	60.7 ± 39.7		48.7 ± 31.7		0.37
Months since diagnosis					0.12
● ≤12 months		0		1	
● 13 to 24 months		5		1	
● 25 to 36 months		2		3	
● 49 to 60 months		0		3	
● ≥61 months		9		6	
Diagnosed breast cancer stage					0.68
● Stage 0		3		1	
● Stage 1		4		5	
● Stage 2		7		5	
● Stage 3		2		3	
Treatment type					0.64
● Surgery only		2		4	
● Surgery + radiation		2		1	
● Surgery + chemo		5		5	
● Surgery + radiation + chemo		7		4	
Tamoxifen use					0.92
● Yes		10		9	
● No		6		5	
Follow-up care in past 12 months					1.00
● Yes		16		14	
● No		0		0	
Clinical breast exam frequency					0.57
● Never		1		0	
● Every 3–6 months		2		2	
● Every 6–12 months		5		5	
● Once yearly		6		7	
● Other		2		0	
Comorbidities					0.23
● None		13		14	
● 1		1		0	
● ≥2		2		0	

* Intent-to-treat analysis presented. Avg. = average; *M* = Mean; SD = Standard Deviation; Freq. = Frequency.

**Table 2:** Baseline comparisons for participants’ primary and secondary outcomes *^,a^.

	Experimental (*n* = 16)	Comparison (*n* = 14)	*p*-Value
	Primary Outcomes		
Daily MVPA	26.7 ± 18.4	20.9 ± 17.6	0.40
Daily LPA	72.9 ± 44.1	77.3 ± 48.2	0.80
Daily SB	378.0 ± 192.5	361.7 ± 201.0	0.82
Daily EE	272.5 ± 166.6	303.1 ± 240.9	0.69
Daily steps	4099.8 ± 2651.6	3092.7 ± 2214.0	0.27
	**Secondary Outcomes**		
	Physiological variables		
Weight (kg)	76.0 ± 13.0	85.0 ± 24.9	0.24
Body fat (%)	39.4 ± 5.5	38.6 ± 9.8	0.81
Cardiorespiratory fitness	113.2 ± 20.7	106.1 ± 23.4	0.39
	Psychosocial variables		
Self-efficacy ^#^	73.3 ± 22.1	80.3 ± 14.5	0.33
Social support ^$^	2.8 ± 1.1	2.1 ± 1.0	0.06
Enjoyment ^$^	3.2 ± 0.5	3.3 ± 0.5	0.55
Barriers ^@^	2.0 ± 0.5	1.9 ± 0.4	0.57
Outcome expectancy ^$^	3.9 ± 0.5	4.1 ± 0.5	0.55
	Quality of life variables		
Physical functioning **	1.2 ± 0.4	1.3 ± 0.3	0.64
Anxiety **	1.8 ± 0.8	1.5 ± 0.7	0.34
Depression **	1.3 ± 0.3	1.1 ± 0.3	0.21
Fatigue **	2.5 ± 1.1	2.3 ± 0.6	0.51
Sleep quality **	3.1 ± 1.0	3.4 ± 0.9	0.39
Sleep disturbances **	2.9 ± 0.6	2.6 ± 0.5	0.17
Social roles/activities limitations **	2.2 ± 1.1	2.1 ± 0.8	0.76
Pain limitations **	1.7 ± 0.8	1.5 ± 0.6	0.51
Pain intensity ^&^	2.0 ± 1.3	2.2 ± 1.9	0.72

* All values Mean ± Standard Deviation; ^a^ Intent-to-treat analysis presented; ^#^ Evaluated on a percentage confidence scale from 0% (Not confident at all) to 100% (Extremely confident); ^$^ Evaluated on 5-point Likert-type scale; ^@^ Evaluated on 4-point Likert-type scale; ** Evaluated on a 5-point Likert-type scale; ^&^ Evaluated on a scale from 0 (no pain) to 10 (worst pain imaginable).

**Table 3:** Descriptive statistics for physical activity-related outcomes by group at baseline and 10 weeks *^,a^.

	Experimental (*n* = 12)		Comparison (*n* = 8)		*p*-Value ^b^
	Baseline	10 Weeks	Baseline	10 Weeks	
Daily MVPA	30.7 ± 13.2	34.2 ± 18.7	30.2 ± 16.2	37.8 ± 20.4	0.49
Daily LPA	91.4 ± 28.8	98.9 ± 29.5	100.4 ± 31.6	108.5 ± 47.9	0.76
Daily SB	464.4 ± 50.7	466.8 ± 34.7	449.2 ± 54.9	449.6 ± 53.2	0.82
Daily EE	333.1 ± 113.3	359.9 ± 147.4	395.5 ± 229.9	418.0 ± 188.9	0.44
Daily steps	4832.4 ± 1816.4	5175.1 ± 2308.2	4411.6 ± 1624.7	4746.0 ± 2044.9	0.76

* All values Mean ± Standard Deviation; ^a^ Per-protocol analysis presented; ^b^ *p*-value represents group difference in change from baseline to 10 weeks for a given outcome as assessed via Mann–Whitney U tests.

**Table 4:** Descriptive statistics for physiological and psychosocial variables by group at baseline and 10 weeks *^,a^.

	Experimental (*n* = 12)		Comparison (*n* = 8)		*p*-Value ^b^
	Baseline	10 Weeks	Baseline	10 Weeks	
	Physiological variables				
Weight (kg)	76.6 ± 13.3	76.9 ± 12.2	78.0 ± 22.6	78.0 ± 23.0	0.62
Body fat (%)	39.8 ± 6.0	40.2 ± 5.4	36.0 ± 10.6	35.0 ± 10.8	0.12
Cardiorespiratory fitness	110.4 ± 18.5	105.7 ± 21.7	104.8 ± 29.3	100.3 ± 21.6	0.97
	**Psychosocial variables**				
Self-efficacy ^#^	75.6 ± 25.1	67.9 ± 26.5	78.2 ± 12.1	71.8 ± 14.8	0.98
Social support ^$^	3.0 ± 1.1	2.7 ± 1.3	2.4 ± 1.1	3.0 ± 1.1	0.05
Enjoyment ^$^	3.3 ± 0.5	3.2 ± 0.5	3.2 ± 0.6	3.3 ± 0.6	0.53
Barriers ^@^	2.0 ± 0.5	2.0 ± 0.5	2.1 ± 0.2	1.8 ± 0.4	0.04
Outcome expectancy ^$^	4.1 ± 0.5	3.9 ± 0.5	4.0 ± 0.6	4.0 ± 0.6	0.34

* All values Mean ± Standard Deviation; ^a^ Per-protocol analysis presented; ^b^ *p*-value represents group difference in change from baseline to 10 weeks for a given outcome as assessed via independent *t*-tests; ^#^ Evaluated on a percentage confidence scale from 0% (Not confident at all) to 100% (Extremely confident); ^$^ Evaluated on 5-point Likert-type scale; ^@^ Evaluated on 4-point Likert-type scale.

**Table 5:** Descriptive statistics for quality of life outcomes by group at baseline and 10 weeks *^,a^.

	Experimental (*n* = 12)		Comparison (*n* = 8)		*p*-Value ^b^
	Baseline	10 Weeks	Baseline	10 Weeks	
Physical functioning **	1.1 ± 0.2	1.1 ± 0.2	1.2 ± 0.2	1.1 ± 0.2	0.78
Anxiety **	1.8 ± 0.8	2.0 ± 0.8	1.7 ± 0.7	1.5 ± 0.7	0.19
Depression **	1.2 ± 0.3	1.4 ± 0.4	1.1 ± 0.3	1.1 ± 0.1	0.41
Fatigue **	2.3 ± 1.0	2.3 ± 0.8	2.4 ± 0.6	2.2 ± 0.9	0.31
Sleep quality **	3.1 ± 0.9	3.3 ± 0.6	3.6 ± 0.9	3.5 ± 0.9	0.62
Sleep disturbances **	2.8 ± 0.6	2.5 ± 0.4	2.5 ± 0.5	2.5 ± 0.4	0.64
Social roles/activities limitations **	2.0 ± 0.9	1.8 ± 0.7	1.9 ± 0.7	1.9 ± 0.5	0.64
Pain limitations **	1.5 ± 0.5	1.5 ± 0.6	1.4 ± 0.5	1.4 ± 0.4	1.0
Pain intensity ^&^	1.8 ± 1.0	1.8 ± 1.8	2.0 ± 1.9	1.8 ± 1.4	0.97

* All values Mean ± Standard Deviation; ^a^ Per-protocol analysis presented; ^b^ *p*-value represents group difference in change from baseline to 10 weeks for a given outcome as assessed via Mann–Whitney U tests; ** Evaluated on a 5-point Likert-type scale; ^&^ Evaluated on a scale from 0 (no pain) to 10 (worst pain imaginable).

## Supplementary Materials

The following are available online at http://www.mdpi.com/2077-0383/7/6/140/s1.
