# Supplementary material for: Effectiveness of Combined Smartwatch and Social Media Intervention on Breast Cancer Survivor Health Outcomes: A 10-Week Pilot Randomized Trial
Source: J Clin Med. 2018 Jun 7;7(6):140. doi: 10.3390/jcm7060140 (PMC6025572; doi:10.3390/jcm7060140)

Supplementary Materials 1. Twice-weekly tips.

The following tips were provided twice a week (once on Monday and once on Thursday) for 10 weeks to all study participants. Psychosocial constructs targeted with each posting are listed in ***bold italics*** *following each tip.*

Week 1

Monday: Did you know the American College of Sports Medicine states that the recommended 30 minutes a day of physical activity can be accumulated in 3 ten-minute bouts?! If you have an extra ten minutes, try going for a brisk walk or bike ride. ***Social-cognitive belief(s) targeted: Promoting Self-Efficacy, Decreasing Barriers***

Thursday: Taking the stairs instead of the elevator is a great way to interject more physical activity into your day! Trying doing so today! ***Social-cognitive belief(s) targeted: Promoting Self-Efficacy***

Week 2

Monday: Did you know that burning an extra 100 calories per day or reducing calorie intake by 100 calories a day can result in weight loss of over 10 pounds in one year?! This can be as simple as a one mile walk after dinner or foregoing a can of soda in exchange for water during the day. Baby steps! ***Social-cognitive belief(s) targeted: Promoting Self-Efficacy, Improving Outcome Expectancy, Decreasing Barriers***

Thursday: Travelling and not confident about your ability to work out? Try one of two things. First, ask the hotel if they have a fitness room or exclusive access to a nearby gym. Note the hours of operation for either and build at least 30 minutes into each day of your vacation to get a short workout in. Second, if you find the hotel does not have a fitness center or access to a nearby gym, explore nearby walking trails or maybe even walk laps around the hotel for the hotel for 30 minutes. Still want to lift? Use your luggage as weights for resistance training. ***Social-cognitive belief(s) targeted: Promoting Self-Efficacy, Decreasing Barriers***

Week 3

Monday: Social support is crucial to continued physical activity engagement. Thus, try to find a good friend willing to go "sweat it out" with you two or three times per week. Although your friend may not be able to join you for every workout, s/he may be able to provide company on the days where you are lacking the motivation to get to the gym. ***Social-cognitive belief(s) targeted: Enhancing Social Support***

Thursday: School work or your job stressing you out? Lucky for you, physical activity releases stress-reducing hormones such as endorphins into the bloodstream even during short 10-minute bouts of exercise. All the more reason to set aside a little time each day to be physically active! ***Social-cognitive belief(s) targeted: Improving Outcome Expectancy***

Week 4

Monday: We schedule dentist appointments, haircuts, and meals. Why not schedule physical activity into your day? Make exercise a part of your daily schedule at a time where physical activity can help you get ready for the day (for morning exercisers) or unwind from the day (for evening exercisers). Treat this scheduled exercise as important and much needed "you" time--allowing you to be better for those around you. Further, you can be confident that you are going to get your workout in when viewing it in this manner. ***Social-cognitive belief(s) targeted: Promoting Self-Efficacy***

Thursday: Worried about your motivation to exercise in the morning or that you will forget your exercise clothes as you head out the door? Place your workout clothes/shoes in front of the door you exit each

morning. In this manner, you will have to move the clothes prior to opening the door, acting as a reminder to be a little more physically active during the day or to not skip the gym in the evening. ***Social-cognitive belief(s) targeted: Promoting Self-Efficacy, Decreasing Barriers***

Week 5

Monday: Setting realistic, yet challenging goals can be a great way to sustain motivation to remain physically active. For example, set the goal of increasing the distance you walk by one-quarter mile each week until you reach three miles. At three miles, consider a walk-run pattern wherein you walk for one minute and then jog for one minute. You can repeat this pattern for a designated amount of time (e.g., 30 minutes) or for a certain distance (e.g., 3 miles). As it gets easier, gradually introduce more jogging and less walking. ***Social-cognitive belief(s) targeted: Promoting Self-Efficacy***

Thursday: Blisters and chaffing caused by the wrong workout clothes/shoes can be a serious threat to continued participation in physical activity. Thus, consider an investment in proper (and good-looking) athletic clothes and shoes. Doing so may just help increase your motivation to be physically active while also decreasing the likelihood of experiencing painful skin irritation! ***Social-cognitive belief(s) targeted: Decreasing Barriers, Improving Outcome Expectancy***

Week 6

Monday: Again, social support is important to physical activity participation. If you are engaging in a new physical activity program, perhaps tell your family and close friends about your new program. Stating your plans out loud not only increases the likelihood that you will continue this physical activity program, but your family and friends will surely ask about it at some point in the future meaning you may be held accountable for sticking to this program! ***Social-cognitive belief(s) targeted: Enhancing Social Support***

Thursday: Water is vital. Although recommendations put forth my numerous health organizations such as the Centers for Disease Control and Prevention and the American College of Sports Medicine state 6-8 cups a day is needed, this does not always hold true for all individuals. Therefore, if you are not confident in your ability to drink enough water, buy a good water bottle that can be used at the gym and during the day and drink each time you feel thirsty. Further, drinking consistently throughout the day can help decrease your appetite and improve digestion, helping you lose weight. ***Social-cognitive belief(s) targeted: Promoting Self-Efficacy, Improving Outcome Expectancy***

Week 7

Monday: One of the best ways to ensure you continue to participate in a physical activity program is finding an activity you enjoy. If you are an individual who prefers to workout alone, perhaps swimming, running, or biking suits you the best. For individuals who prefer to workout with others, consider group exercise classes such as yoga or step aerobics such as Zumba or dancing. Additionally, do not be afraid to mix and match different types of exercise! If you are going to sweat you might as well be doing something that interests you! ***Social-cognitive belief(s) targeted: Increasing Enjoyment, Enhancing Social Support***

Thursday: Face it, you have put in the time in the gym and, perhaps, even lost a little weight in the process. Consider a monthly reward. This reward can be anything from the purchase of that one shirt that you have been dying to add to your wardrobe to a night out with your significant other. Yet, whatever the reward is, make sure that it does not derail your quest for better health and participation in physical activity. ***Social-cognitive belief(s) targeted: Improving Outcome Expectancy***

Week 8

Monday: Did you know that physical activity has been linked to greater feelings of well-being? Well, it has! Physical activity, even in bouts as short as ten minutes can increase "good" hormones within the body such as endorphins. Indeed, release of these hormones on a regular basis as a result of continued physical activity participation has been found to lower likelihood of diseases such as depression and increase self-esteem. ***Social-cognitive belief(s) targeted: Improving Outcome Expectancy***

Thursday: Sitting at a desk all day is not healthy. Consider setting your watch or phone to beep every thirty minutes during the day at which point you HAVE to get up and go for a five minute walk or engage in some light stretching. Not only will this give your body a much needed boost, it might also give your mind the break in concentration it needs and allow you to be more productive while doing homework or completing work for your job. ***Social-cognitive belief(s) targeted: Improving Outcome Expectancy***

Week 9

Monday: Ensure you do not pull a muscle during your workout. Start each exercise session with some light exercise such as jumping jacks, brisk walking, or light biking/weight lifting. This will allow you to heat your core temperature up to a point wherein physical activity engagement does not pose much risk to your body. ***Social-cognitive belief(s) targeted: Decreasing Barriers***

Thursday: Static stretching prior to exercise may actually decrease workout performance and, due to the fact the muscles are not warm, is not increasing flexibility. However, engaging in static stretching after engaging in physical activity is one of the best ways to increase flexibility and may even help with delayed-onset muscle soreness. Ensure your stretching routine is sufficient to stretch all major muscles of the upper and lower body. Further, only stretch to the point of slight discomfort, not pain, and hold the stretch for 20 seconds. ***Social-cognitive belief(s) targeted: Promoting Self-Efficacy, Improving Outcome Expectancy***

Week 10

Monday: Sleep may not seem important to physical activity, but it is. Not getting enough sleep is a sure-fire way to experience decreases in motivation for engaging in physical activity. Therefore, aim for 6 to 8 hours of sleep each night. Moreover, try to cut out all screen time in the 10 to 15 minutes prior to going to sleep as watching TV or using your computer/smartphone to cruise social media or read the news decreases the body's ability to produce melatonin, a key sleep hormone. Finally, consider removing any TV from the bedroom and/or not playing music while you sleep as this background noise can actually decrease sleep quality. Making these small changes can go a long way in helping you feel more rested and ready to engage in physical activity (and life) the next day! ***Social-cognitive belief(s) targeted: Promoting Self-Efficacy, Decreasing Barriers***

Thursday: Crank those tunes! If music is something you know will get you motivated to engage in your workout, consider investing in some athletic-oriented headphones that are sweat resistant and capable of staying in the ear during exercise. Keeping an up-to-date playlist of your favorite songs will allow you to have a better workout. This is especially true of exercisers preferring to exercise indoors. However, if exercising outdoors, consider leaving the headphones at home and enjoying the scenery as use of headphones while exercising outdoors can put you in danger. ***Social-cognitive belief(s) targeted: Increasing Enjoyment***

Supplementary Materials 2. Exercise made easy: beginners programming.

Below is a 10-week outline (40 workouts) of exercise programming for individuals with little experience in the gym or those just coming back from a long time away from the gym. As such, this workout program is programmed for exercise 4 times a week for roughly an hour, with simple exercises placed within. Notably, under each of the main exercises for each day are **suggested alternative exercises** which may better appeal to breast cancer survivors recovering from treatment.

Finally, for each cardio workout listed below, you will use the Rating of Perceived Exertion (RPE) scale to gauge your intensity level. This scale is listed below, allowing for comprehension of the intensity level each cardio workout should be completed at.

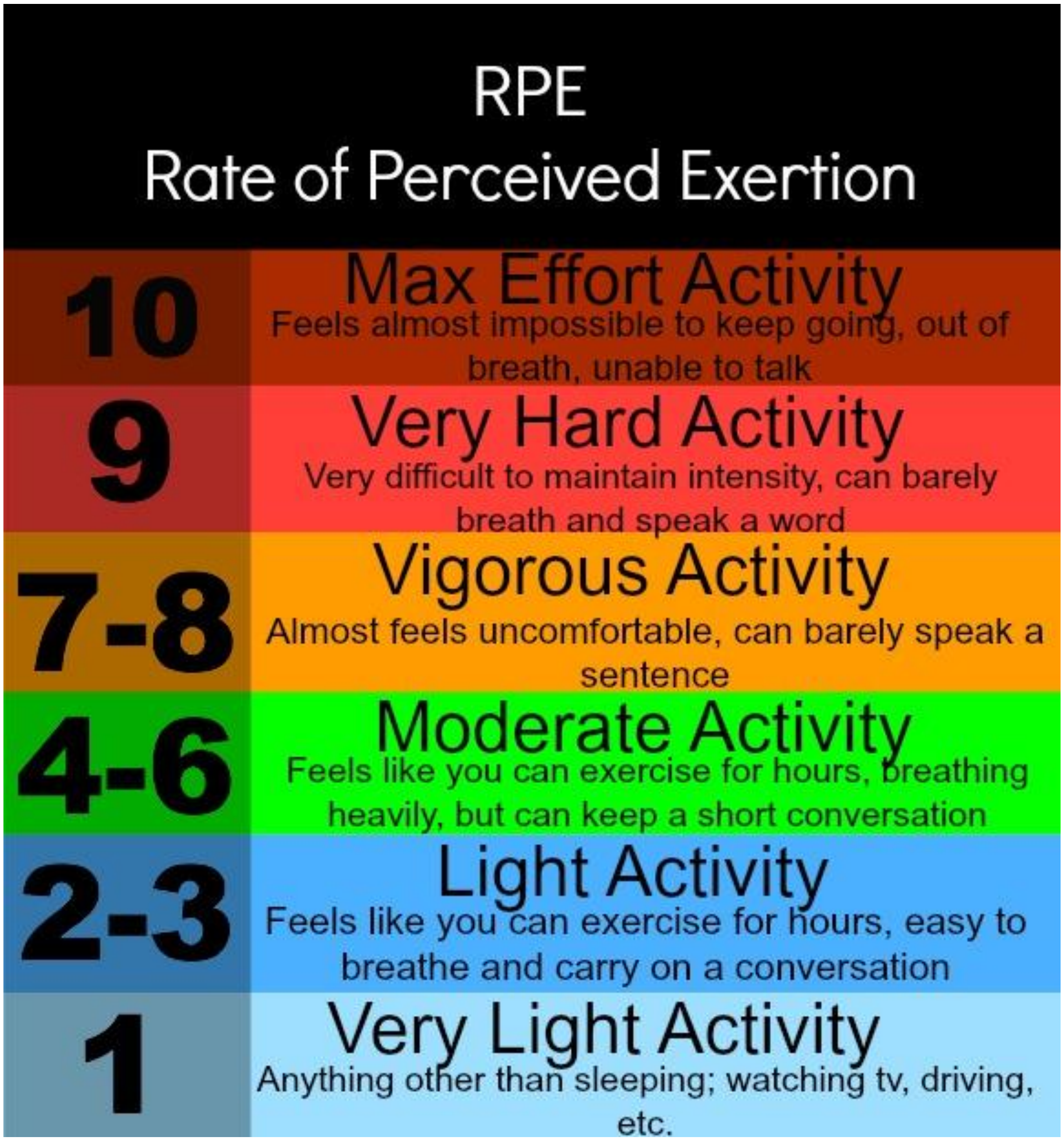

<u>Month 1: Endurance Phase</u>

For each strength exercise, the endurance phase of this program will include higher numbers of repetitions (10-12 repetitions for each set) for each exercise with lower weight. You should rest for 60 seconds to 1.5 minutes between each strength exercise. ***A Diagrammatic explanation for each exercise is available starting on Page 20 of this workout program.*** **For each cardio workout,** feel free to select your favorite cardio exercise. Some cardio exercise options include, but are not limited to: walking, jogging, running, biking, swimming, stairclimbing, rowing, elliptical exercise, etc.

<u>Week 1</u>
Day 1:

- **Strength Workout**
    - **Dumbbell Lunges (count every other leg):** 3 sets x 10-12 repetitions
        - **ALTERNATIVE: Walking Lunges without Dumbbells (still count every other leg)**
    - **Hamstring Curls:** 3 sets x 10-12 repetitions
        - **ALTERNATIVE: Glute Kickbacks**
    - **Calf Raises:** 3 sets x 50 repetitions
        - **ALTERNATIVE: Seated Heel Lifts**
    - **Bicycle Crunches:** 2 sets x 50 revolutions
        - **ALTERNATIVE: Standard Crunches**
- **Cardio Workout**
    - **30 minutes selected cardio exercise at moderate pace:** RPE Scale: 4-6

Day 2:

- **Strength Workout**
    - **Dumbbell Bench Press:** 3 sets x 10-12 repetitions
        - **ALTERNATIVE: Push-Ups on Knees (only go down as far as your mobility will allow)**
    - **Tricep Kickbacks:** 3 sets x 10-12 repetitions
        - **ALTERNATIVE: Tricep Dips on Chair (only go down as far as your mobility will allow)**
    - **Dumbbell Chest Flies (go light and pretend to "hug a tree"):** 3 sets x 10-12 repetitions
        - **ALTERNATIVE: Wide-Grip Wall Push-Ups**
    - **Bicycle Crunches:** 2 sets x 50 revolutions
        - **ALTERNATIVE: Standard Crunches**
- **Cardio Workout**
    - **30 minutes selected cardio exercise high-intensity intervals:** These intervals will be comprised of 1 minute EASY (RPE Scale: 2-3) and 1 minute HARD (RPE Scale: 7-8) and repeated until time is up.

Day 3:

- **Strength Workout**
    - **Upright Rows:** 3 sets x 10-12 repetitions
        - **ALTERNATIVE: Forward Arm Raises**
    - **One-Arm Rows (pretend you are "starting a lawnmower"):** 3 sets x 10-12 repetitions

        - **ALTERNATIVE: Single Arm Circles**
    - **Lat Pulldowns:** 3 sets x 10-12 repetitions
        - **ALTERNATIVE: Seated Reaching Exercise (count every other side)**
    - **Dumbbell Shrugs:** 2 sets x 10-12 repetitions
        - **ALTERNATIVE: Shoulders to Ears**
    - **Sit-ups:** 2 sets x 25 repetitions
        - **ALTERNATIVE: Standard Crunches**
- **Cardio Workout**
    - **30 minutes selected cardio exercise at moderate pace:** RPE Scale: 4-6

Day 4:

- **Cardio Workout ONLY**
    - **60 minutes selected cardio at light/moderate pace:** RPE Scale: 3-6

<u>Week 2</u>
Day 5:

- **Strength Workout**
    - **Goblet Squats with Dumbbell:** 3 sets x 10-12 repetitions
        - **ALTERNATIVE: Air Squats**
    - **Hamstring Curls:** 3 sets x 10-12 repetitions
        - **ALTERNATIVE: Glute Kickbacks**
    - **Calf Raises:** 3 sets x 50 repetitions
        - **ALTERNATIVE: Seated Heel Lifts**
    - **Leg Raises:** 2 sets x 25 raises
        - **ALTERNATIVE: Seated Leg Lifts**
- **Cardio Workout**
    - **30 minutes selected cardio exercise at moderate pace:** RPE Scale: 4-6

Day 6:

- **Strength Workout**
    - **Incline Dumbbell Bench Press:** 3 sets x 10-12 repetitions
        - **ALTERNATIVE: Push-Ups on Knees (only go down as far as your mobility will allow)**
    - **Diamond Push-Ups:** 3 sets x 10-12 repetitions
        - **ALTERNATIVE: Close-Grip Wall Push-Ups**
    - **Dumbbell Chest Flies (go light and pretend to "hug a tree"):** 3 sets x 10-12 repetitions
        - **ALTERNATIVE: Wide-Grip Wall Push-Ups**
    - **Jack Knives:** 2 sets x 20 repetitions
        - **ALTERNATIVE: Standard Crunches**
- **Cardio Workout**
    - **30 minutes selected cardio exercise high-intensity intervals:** These intervals will be comprised of 1 minute EASY (RPE Scale: 2-3) and 1 minute HARD (RPE Scale: 7-8) and repeated until time is up.

Day 7:

- **Strength Workout**
  - **Upright Rows:** 3 sets x 10-12 repetitions
    - **ALTERNATIVE: Forward Arm Raises**
  - **One-Arm Rows (pretend you are "starting a lawnmower"):** 3 sets x 10-12 repetitions
    - **ALTERNATIVE: Single Arm Circles**
  - **Lat Pulldowns:** 3 sets x 10-12 repetitions
    - **ALTERNATIVE: Seated Reaching Exercise (count every other side)**
  - **Seated Cable Row:** 2 sets x 10-12 repetitions
    - **ALTERNATIVE: Single Arm Circles**
  - **Sit-ups:** 2 sets x 25 repetitions
    - **ALTERNATIVE: Standard Crunches**
- **Cardio Workout**
  - **30 minutes selected cardio exercise at moderate pace:** RPE Scale: 4-6

Day 8:

- **Cardio Workout ONLY**
  - **60 minutes selected cardio at light/moderate pace:** RPE Scale: 3-6

Week 3
Day 9:

- **Strength Workout**
  - **Dumbbell Lunges (count every other leg):** 3 sets x 10-12 repetitions
    - **ALTERNATIVE: Walking Lunges without Dumbbells (still only count every other leg)**
  - **Hamstring Curls:** 3 sets x 10-12 repetitions
    - **ALTERNATIVE: Glute Kickbacks**
  - **Calf Raises:** 3 sets x 50 repetitions
    - **ALTERNATIVE: Seated Heel Lifts**
  - **Bicycle Crunches:** 2 sets x 50 revolutions
    - **ALTERNATIVE: Standard Crunches**
- **Cardio Workout**
  - **30 minutes selected cardio exercise at moderate pace:** RPE Scale: 4-6

Day 10:

- **Strength Workout**
  - **Dumbbell Bench Press:** 3 sets x 10-12 repetitions
    - **ALTERNATIVE: Push-Ups on Knees (only go down as far as your mobility will allow)**
  - **Tricep Kickbacks:** 3 sets x 10-12 repetitions
    - **ALTERNATIVE: Tricep Dips on Chair (only go down as far as your mobility will allow)**
  - **Dumbbell Chest Flies (go light and pretend to "hug a tree"):** 3 sets x 10-12 repetitions
    - **ALTERNATIVE: Wide-Grip Wall Push-Ups**
  - **Bicycle Crunches:** 2 sets x 50 revolutions

        - **ALTERNATIVE: Standard Crunches**
- **Cardio Workout**
    - **30 minutes selected cardio exercise high-intensity intervals:** These intervals will be comprised of 1 minute EASY (RPE Scale: 2-3) and 1 minute HARD (RPE Scale: 7-8) and repeated until time is up.

Day 11:

- **Strength Workout**
    - **Upright Rows:** 3 sets x 10-12 repetitions
        - **ALTERNATIVE: Forward Arm Raises**
    - **One-Arm Rows (pretend you are "starting a lawnmower"):** 3 sets x 10-12 repetitions
        - **ALTERNATIVE: Single Arm Circles**
    - **Lat Pulldowns:** 3 sets x 10-12 repetitions
        - **ALTERNATIVE: Seated Reaching Exercise (count every other side)**
    - **Dumbbell Shrugs:** 2 sets x 10-12 repetitions
        - **ALTERNATIVE: Shoulders to Ears**
    - **Sit-ups:** 2 sets x 25 repetitions
        - **ALTERNATIVE: Standard Crunches**
- **Cardio Workout**
    - **30 minutes selected cardio exercise at moderate pace:** RPE Scale: 4-6

Day 12:

- **Cardio Workout ONLY**
    - **60 minutes selected cardio at light/moderate pace:** RPE Scale: 3-6

<u>Week 4</u>
Day 13:

- **Strength Workout**
    - **Goblet Squats with Dumbbell:** 3 sets x 10-12 repetitions
        - **ALTERNATIVE: Air Squats**
    - **Hamstring Curls:** 3 sets x 10-12 repetitions
        - **ALTERNATIVE: Glute Kickbacks**
    - **Calf Raises:** 3 sets x 50 repetitions
        - **ALTERNATIVE: Seated Heel Lifts**
    - **Leg Raises:** 2 sets x 25 raises
        - **ALTERNATIVE: Seated Leg Lifts**
- **Cardio Workout**
    - **30 minutes selected cardio exercise at moderate pace:** RPE Scale: 4-6

Day 14:

- **Strength Workout**
    - **Incline Dumbbell Bench Press:** 3 sets x 10-12 repetitions
        - **ALTERNATIVE: Push-Ups on Knees (only go down as far as your mobility will allow)**
    - **Diamond Push-Ups:** 3 sets x 10-12 repetitions

        - **ALTERNATIVE: Close-Grip Wall Push-Ups**
    - **Dumbbell Chest Flies (go light and pretend to "hug a tree"):** 3 sets x 10-12 repetitions
        - **ALTERNATIVE: Wide-Grip Wall Push-Ups**
    - **Jack Knives:** 2 sets x 20 repetitions
        - **ALTERNATIVE: Standard Crunches**
- **Cardio Workout**
    - **30 minutes selected cardio exercise high-intensity intervals:** These intervals will be comprised of 1 minute EASY (RPE Scale: 2-3) and 1 minute HARD (RPE Scale: 7-8) and repeated until time is up.

Day 15:

- **Strength Workout**
    - **Upright Rows:** 3 sets x 10-12 repetitions
        - **ALTERNATIVE: Forward Arm Raises**
    - **One-Arm Rows (pretend you are "starting a lawnmower"):** 3 sets x 10-12 repetitions
        - **ALTERNATIVE: Single Arm Circles**
    - **Lat Pulldowns:** 3 sets x 10-12 repetitions
        - **ALTERNATIVE: Seated Reaching Exercise (count every other side)**
    - **Seated Cable Row:** 2 sets x 10-12 repetitions
        - **ALTERNATIVE: Single Arm Circles**
    - **Sit-ups:** 2 sets x 25 repetitions
        - **ALTERNATIVE: Standard Crunches**
- **Cardio Workout**
    - **30 minutes selected cardio exercise at moderate pace:** RPE Scale: 4-6

Day 16:

- **Cardio Workout ONLY**
    - **60 minutes selected cardio at light/moderate pace:** RPE Scale: 3-6

<u>Month 2: Strength Phase</u>

For each strength exercise, the strength phase of this program will include a moderate number of repetitions (7-9 repetitions for each set) for each exercise with moderate weight. You should rest for 1.5 to 2.5 minutes between each strength exercise. ***A Diagrammatic explanation for each exercise is available starting on Page 20 of this workout program.*** **For each cardio workout,** feel free to select your favorite cardio exercise. Some cardio exercise options include, but are not limited to: walking, jogging, running, biking, swimming, stairclimbing, rowing, elliptical exercise, etc.

<u>Week 5</u>
Day 17:

- **Strength Workout**
    - **Dumbbell Lunges (count every other leg):** 3 sets x 7-9 repetitions
        - **ALTERNATIVE: Walking Lunges without Dumbbells (still count every other leg)**

- **Hamstring Curls:** 3 sets x 7-9 repetitions
  - **ALTERNATIVE: Glute Kickbacks**
- **Calf Raises:** 3 sets x 50 repetitions
  - **ALTERNATIVE: Seated Heel Lifts**
- **Bicycle Crunches:** 2 sets x 50 revolutions
  - **ALTERNATIVE: Standard Crunches**

- **Cardio Workout**
  - **30 minutes selected cardio exercise at moderate pace:** RPE Scale: 4-6

Day 18:

- **Strength Workout**
  - **Dumbbell Bench Press:** 3 sets x 7-9 repetitions
    - **ALTERNATIVE: Push-Ups on Knees (only go down as far as your mobility will allow)**
  - **Tricep Kickbacks:** 3 sets x 7-9 repetitions
    - **ALTERNATIVE: Tricep Dips on Chair (only go down as far as your mobility will allow)**
  - **Dumbbell Chest Flies (go light and pretend to "hug a tree"):** 3 sets x 7-9 repetitions
    - **ALTERNATIVE: Wide-Grip Wall Push-Ups**
  - **Bicycle Crunches:** 2 sets x 50 revolutions
    - **ALTERNATIVE: Standard Crunches**
- **Cardio Workout**
  - **30 minutes selected cardio exercise high-intensity intervals:** These intervals will be comprised of 1 minute EASY (RPE Scale: 2-3) and 1 minute HARD (RPE Scale: 7-8) and repeated until time is up.

Day 19:

- **Strength Workout**
  - **Upright Rows:** 3 sets x 7-9 repetitions
    - **ALTERNATIVE: Forward Arm Raises**
  - **One-Arm Rows (pretend you are "starting a lawnmower"):** 3 sets x 7-9 repetitions
    - **ALTERNATIVE: Single Arm Circles**
  - **Lat Pulldowns:** 3 sets x 7-9 repetitions
    - **ALTERNATIVE: Seated Reaching Exercise (count ever other side)**
  - **Dumbbell Shrugs:** 2 sets x 7-9 repetitions
    - **ALTERNATIVE: Shoulders to Ears**
  - **Sit-ups:** 2 sets x 25 repetitions
    - **ALTERNATIVE: Standard Crunches**
- **Cardio Workout**
  - **30 minutes selected cardio exercise at moderate pace:** RPE Scale: 4-6

Day 20:

- **Cardio Workout ONLY**
  - **60 minutes selected cardio at light/moderate pace:** RPE Scale: 3-6

<u>**Week 6**</u>
Day 21:

- **Strength Workout**
  - **Goblet Squats with Dumbbell:** 3 sets x 7-9 repetitions
    - **ALTERNATIVE: Air Squats**
  - **Hamstring Curls:** 3 sets x 7-9 repetitions
    - **ALTERNATIVE: Glute Kickbacks**
  - **Calf Raises:** 3 sets x 50 repetitions
    - **ALTERNATIVE: Seated Heel Lifts**
  - **Leg Raises:** 2 sets x 25 raises
    - **ALTERNATIVE: Seated Leg Lifts**
- **Cardio Workout**
  - **30 minutes selected cardio exercise at moderate pace:** RPE Scale: 4-6

Day 22:

- **Strength Workout**
  - **Incline Dumbbell Bench Press:** 3 sets x 7-9 repetitions
    - **ALTERNATIVE: Push-Ups on Knees (only go down as far as your mobility will allow)**
  - **Diamond Push-Ups:** 3 sets x 7-9 repetitions
    - **ALTERNATIVE: Close-Grip Wall Push-Ups**
  - **Dumbbell Chest Flies (go light and pretend to "hug a tree"):** 3 sets x 7-9 repetitions
    - **ALTERNATIVE: Wide-Grip Wall Push-Ups**
  - **Jack Knives:** 2 sets x 20 repetitions
    - **ALTERNATIVE: Standard Crunches**
- **Cardio Workout**
  - **30 minutes selected cardio exercise high-intensity intervals:** These intervals will be comprised of 1 minute EASY (RPE Scale: 2-3) and 1 minute HARD (RPE Scale: 7-8) and repeated until time is up.

Day 23:

- **Strength Workout**
  - **Upright Rows:** 3 sets x 7-9 repetitions
    - **ALTERNATIVE: Forward Arm Raises**
  - **One-Arm Rows (pretend you are "starting a lawnmower"):** 3 sets x 7-9 repetitions
    - **ALTERNATIVE: Single Arm Circles**
  - **Lat Pulldowns:** 3 sets x 7-9 repetitions
    - **ALTERNATIVE: Seated Reaching Exercise (count every other side)**
  - **Seated Cable Row:** 2 sets x 7-9 repetitions
    - **ALTERNATIVE: Single Arm Circles**
  - **Sit-ups:** 2 sets x 25 repetitions
    - **ALTERNATIVE: Standard Crunches**
- **Cardio Workout**
  - **30 minutes selected cardio exercise at moderate pace:** RPE Scale: 3-6

Day 24:

- **Cardio Workout ONLY**
  - **60 minutes selected cardio at light/moderate pace:** RPE Scale: 3-6

<u>Week 7</u>
Day 25:

- **Strength Workout**
  - **Dumbbell Lunges (count every other leg):** 3 sets x 7-9 repetitions
    - **ALTERNATIVE: Walking Lunges without Dumbbells (still count every other leg)**
  - **Hamstring Curls:** 3 sets x 7-9 repetitions
    - **ALTERNATIVE: Glute Kickbacks**
  - **Calf Raises:** 3 sets x 50 repetitions
    - **ALTERNATIVE: Seated Heel Lifts**
  - **Bicycle Crunches:** 2 sets x 50 revolutions
    - **ALTERNATIVE: Standard Crunches**
- **Cardio Workout**
  - **30 minutes selected cardio exercise at moderate pace:** RPE Scale: 4-6

Day 26:

- **Strength Workout**
  - **Dumbbell Bench Press:** 3 sets x 7-9 repetitions
    - **ALTERNATIVE: Push-Ups on Knees (only go down as far as your mobility will allow)**
  - **Tricep Kickbacks:** 3 sets x 7-9 repetitions
    - **ALTERNATIVE: Tricep Dips on Chair (only go down as far as your mobility will allow)**
  - **Dumbbell Chest Flies (go light and pretend to "hug a tree"):** 3 sets x 7-9 repetitions
    - **ALTERNATIVE: Wide-Grip Wall Push-Ups**
  - **Bicycle Crunches:** 2 sets x 50 revolutions
    - **ALTERNATIVE: Standard Crunches**
- **Cardio Workout**
  - **30 minutes selected cardio exercise high-intensity intervals:** These intervals will be comprised of 1 minute EASY (RPE Scale: 2-3) and 1 minute HARD (RPE Scale: 7-8) and repeated until time is up.

Day 27:

- **Strength Workout**
  - **Upright Rows:** 3 sets x 7-9 repetitions
    - **ALTERNATIVE: Forward Arm Raises**
  - **One-Arm Rows (pretend you are "starting a lawnmower"):** 3 sets x 7-9 repetitions
    - **ALTERNATIVE: Single Arm Circles**
  - **Lat Pulldowns:** 3 sets x 7-9 repetitions

        - **ALTERNATIVE: Seated Reaching Exercise (count every other side)**
    - **Dumbbell Shrugs:** 2 sets x 7-9 repetitions
        - **ALTERNATIVE: Shoulders to Ears**
    - **Sit-ups:** 2 sets x 25 repetitions
        - **ALTERNATIVE: Standard Crunches**
- **Cardio Workout**
    - **30 minutes selected cardio exercise at moderate pace:** RPE Scale: 4-6

Day 28:

- **Cardio Workout ONLY**
    - **60 minutes selected cardio at light/moderate pace:** RPE Scale: 3-6

<u>Week 8</u>
Day 29:

- **Strength Workout**
    - **Goblet Squats with Dumbbell:** 3 sets x 7-9 repetitions
        - **ALTERNATIVE: Air Squats**
    - **Hamstring Curls:** 3 sets x 7-9 repetitions
        - **ALTERNATIVE: Glute Kickbacks**
    - **Calf Raises:** 3 sets x 50 repetitions
        - **ALTERNATIVE: Seated Heel Lifts**
    - **Leg Raises:** 2 sets x 25 raises
        - **ALTERNATIVE: Seated Leg Lifts**
- **Cardio Workout**
    - **30 minutes selected cardio exercise at moderate pace:** RPE Scale: 4-6

Day 30:

- **Strength Workout**
    - **Incline Dumbbell Bench Press:** 3 sets x 7-9 repetitions
        - **ALTERNATIVE: Push-Ups on Knees (only go down as far as your mobility will allow)**
    - **Diamond Push-Ups:** 3 sets x 7-9 repetitions
        - **ALTERNATIVE: Close-Grip Wall Push-Ups**
    - **Dumbbell Chest Flies (go light and pretend to "hug a tree"):** 3 sets x 7-9 repetitions
        - **ALTERNATIVE: Wide-Grip Wall Push-Ups**
    - **Jack Knives:** 2 sets x 20 repetitions
        - **ALTERNATIVE: Standard Crunches**
- **Cardio Workout**
    - **30 minutes selected cardio exercise high-intensity intervals:** These intervals will be comprised of 1 minute EASY (RPE Scale: 2-3) and 1 minute HARD (RPE Scale: 7-8) and repeated until time is up.

Day 31:

- **Strength Workout**
    - **Upright Rows:** 3 sets x 7-9 repetitions

        - **ALTERNATIVE: Forward Arm Raises**
    - **One-Arm Rows (pretend you are "starting a lawnmower"):** 3 sets x 7-9 repetitions
        - **ALTERNATIVE: Single Arm Circles**
    - **Lat Pulldowns:** 3 sets x 7-9 repetitions
        - **ALTERNATIVE: Seated Reaching Exercise (count every other side)**
    - **Seated Cable Row:** 2 sets x 7-9 repetitions
        - **ALTERNATIVE: Single Arm Circles**
    - **Sit-ups:** 2 sets x 25 repetitions
        - **ALTERNATIVE: Standard Crunches**
- **Cardio Workout**
    - **30 minutes selected cardio exercise at moderate pace:** RPE Scale: 3-6

Day 32:

- **Cardio Workout ONLY**
    - **60 minutes selected cardio at light/moderate pace:** RPE Scale: 3-6

<u>Month 3: Power Phase</u>

For each strength exercise, the power phase of this program will include a lower number of repetitions (4-6 repetitions for each set) for each exercise with higher weight. You should rest for 2.5 to 4.5 minutes between each strength exercise. ***A Diagrammatic explanation for each exercise is available starting on Page 20 of this workout program.*** **For each cardio workout,** feel free to select your favorite cardio exercise. Some cardio exercise options include, but are not limited to: walking, jogging, running, biking, swimming, stairclimbing, rowing, elliptical exercise, etc.

<u>Week 9</u>
Day 33:

- **Strength Workout**
    - **Dumbbell Lunges (count every other leg):** 3 sets x 4-6 repetitions
        - **ALTERNATIVE: Walking Lunges without Dumbbells (still count every other leg)**
    - **Hamstring Curls:** 3 sets x 4-6 repetitions
        - **ALTERNATIVE: Glute Kickbacks**
    - **Calf Raises:** 3 sets x 50 repetitions
        - **ALTERNATIVE: Seated Heel Lifts**
    - **Bicycle Crunches:** 2 sets x 50 revolutions
        - **ALTERNATIVE: Standard Crunches**
- **Cardio Workout**
    - **30 minutes selected cardio exercise at moderate pace:** RPE Scale: 4-6

Day 34:

- **Strength Workout**
    - **Dumbbell Bench Press:** 3 sets x 4-6 repetitions
        - **ALTERNATIVE: Push-Ups on Knees (only go down as far as your mobility will allow)**

  - **Tricep Kickbacks:** 3 sets x 4-6 repetitions
    - **ALTERNATIVE: Tricep Dips on Chair (only go down as far as your mobility will allow)**
  - **Dumbbell Chest Flies (go light and pretend to "hug a tree"):** 3 sets x 4-6 repetitions
    - **ALTERNATIVE: Wide-Grip Wall Push-Ups**
  - **Bicycle Crunches:** 2 sets x 50 revolutions
    - **ALTERNATIVE: Standard Crunches**
- **Cardio Workout**
  - **30 minutes selected cardio exercise high-intensity intervals:** These intervals will be comprised of 1 minute EASY (RPE Scale: 2-3) and 1 minute HARD (RPE Scale: 7-8) and repeated until time is up.

Day 35:

- **Strength Workout**
  - **Upright Rows:** 3 sets x 4-6 repetitions
    - **ALTERNATIVE: Forward Arm Raises**
  - **One-Arm Rows (pretend you are "starting a lawnmower"):** 3 sets x 4-6 repetitions
    - **ALTERNATIVE: Single Arm Circles**
  - **Lat Pulldowns:** 3 sets x 4-6 repetitions
    - **ALTERNATIVE: Seated Reaching Exercise (count every other side)**
  - **Dumbbell Shrugs:** 2 sets x 4-6 repetitions
    - **ALTERNATIVE: Shoulders to Ears**
  - **Sit-ups:** 2 sets x 25 repetitions
    - **ALTERNATIVE: Standard Crunches**
- **Cardio Workout**
  - **30 minutes selected cardio exercise at moderate pace:** RPE Scale: 4-6

Day 36:

- **Cardio Workout ONLY**
  - **60 minutes selected cardio at light/moderate pace:** RPE Scale: 3-6

<u>Week 10</u>
Day 37:

- **Strength Workout**
  - **Goblet Squats with Dumbbell:** 3 sets x 4-6 repetitions
    - **ALTERNATIVE: Air Squats**
  - **Hamstring Curls:** 3 sets x 4-6 repetitions
    - **ALTERNATIVE: Glute Kickbacks**
  - **Calf Raises:** 3 sets x 50 repetitions
    - **ALTERNATIVE: Seated Heel Lifts**
  - **Leg Raises:** 2 sets x 25 raises
    - **ALTERNATIVE: Seated Leg Lifts**
- **Cardio Workout**
  - **30 minutes selected cardio exercise at moderate pace:** RPE Scale: 4-6

Day 38:

- **Strength Workout**
  - **Incline Dumbbell Bench Press:** 3 sets x 4-6 repetitions
    - **ALTERNATIVE: Push-Ups on Knees (only go down as far as your mobility will allow)**
  - **Diamond Push-Ups:** 3 sets x 4-6 repetitions
    - **ALTERNATIVE: Close-Grip Wall Push-Ups**
  - **Dumbbell Chest Flies (go light and pretend to "hug a tree"):** 3 sets x 4-6 repetitions
    - **ALTERNATIVE: Wide-Grip Wall Push-Ups**
  - **Jack Knives:** 2 sets x 20 repetitions
    - **ALTERNATIVE: Standard Crunches**
- **Cardio Workout**
  - **30 minutes selected cardio exercise high-intensity intervals:** These intervals will be comprised of 1 minute EASY (RPE Scale: 2-3) and 1 minute HARD (RPE Scale: 7-8) and repeated until time is up.

Day 39:

- **Strength Workout**
  - **Upright Rows:** 3 sets x 4-6 repetitions
    - **ALTERNATIVE: Forward Arm Raises**
  - **One-Arm Rows (pretend you are "starting a lawnmower"):** 3 sets x 4-6 repetitions
    - **ALTERNATIVE: Single Arm Circles**
  - **Lat Pulldowns:** 3 sets x 4-6 repetitions
    - **ALTERNATIVE: Seated Reaching Exercise (count every other side)**
  - **Seated Cable Row:** 2 sets x 4-6 repetitions
    - **ALTERNATIVE: Single Arm Circles**
  - **Sit-ups:** 2 sets x 25 repetitions
    - **ALTERNATIVE: Standard Crunches**
- **Cardio Workout**
  - **30 minutes selected cardio exercise at moderate pace:** RPE Scale: 4-6

Day 40:

- **Cardio Workout ONLY**
  - **60 minutes selected cardio at light/moderate pace:** RPE Scale: 3-6

__Diagrammatic Explanation of Exercises__

NOTE: Exercises are in alphabetical order with the easier alternative(s) (if available) listed below the main exercises.

Bicycle Crunches:

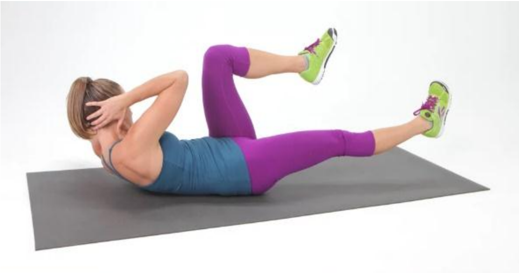

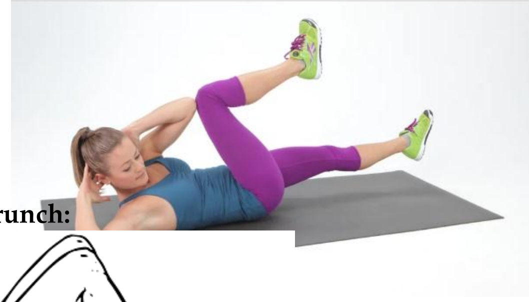

ALTERNATIVE: Standard Crunch:

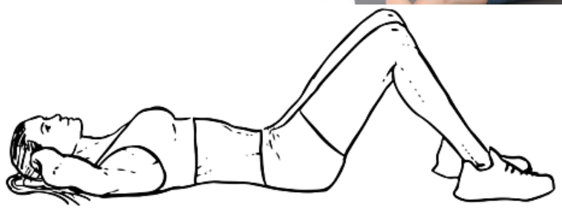

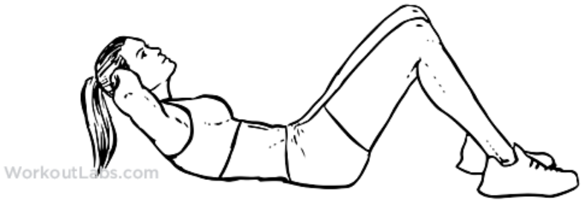

Calf Raises:

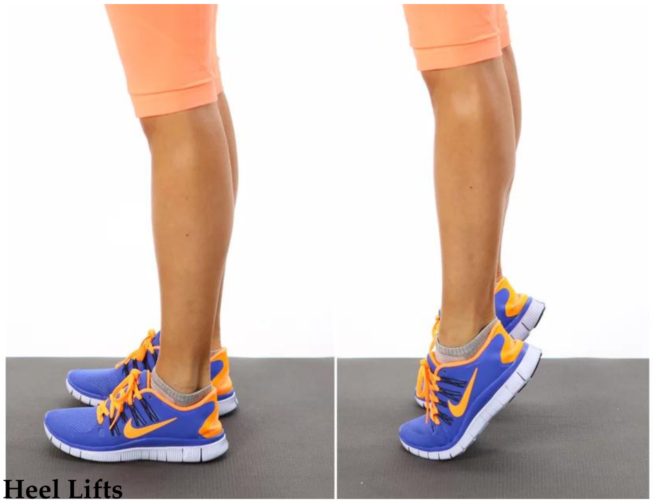

ALTERNATIVE: Seated Heel Lifts

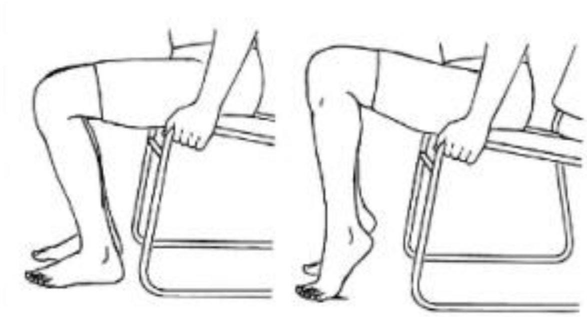

Dumbbell Bench Press:

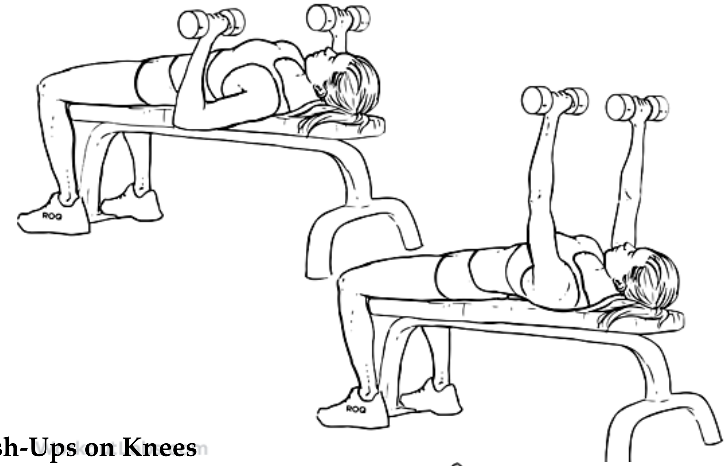

ALTERNATIVE: Push-Ups on Knees

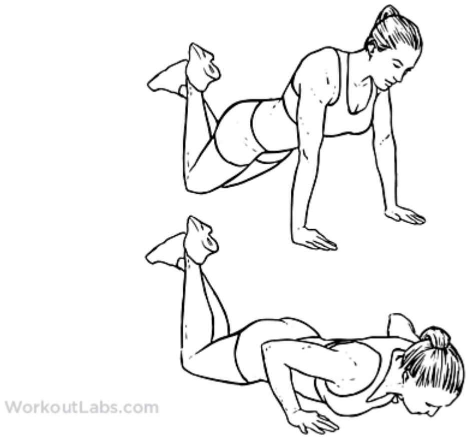

Dumbbell Chest Flies:

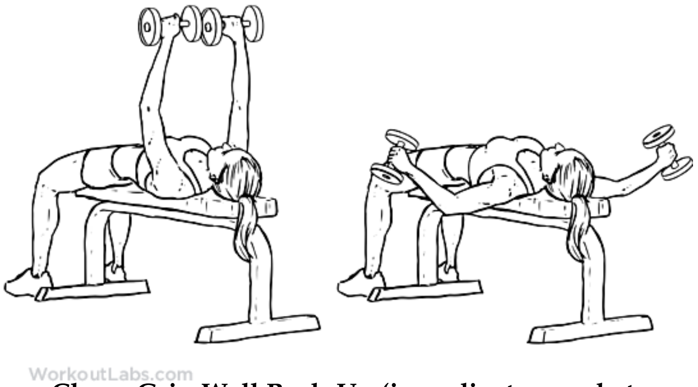

ALTERNATIVE: Wide- or Close- Grip Wall Push-Up (i.e., adjust space between hands with wide-grip meaning hands are further apart and close-grip meaning hands are closer together)

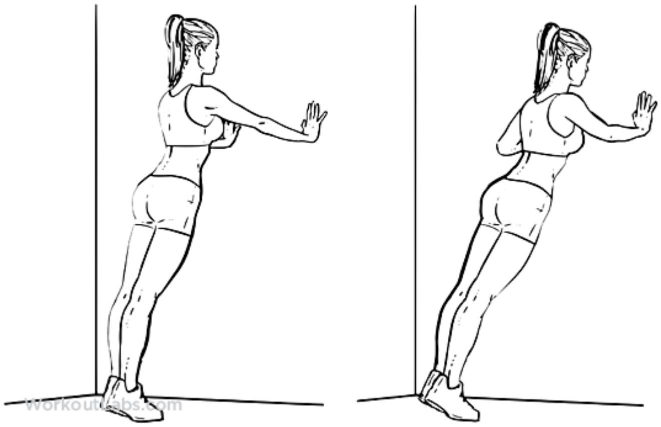

Dumbbell Lunges:

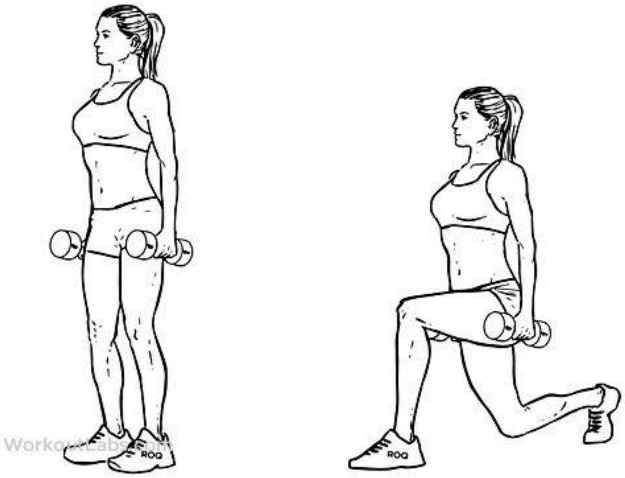

ALTERNATIVE: Walking Lunges without Dumbbells:

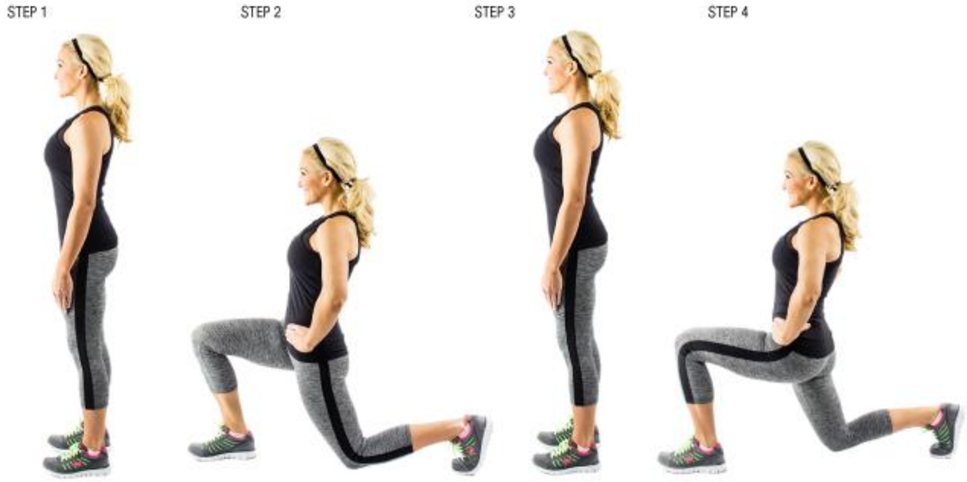

Dumbbell Shrugs:

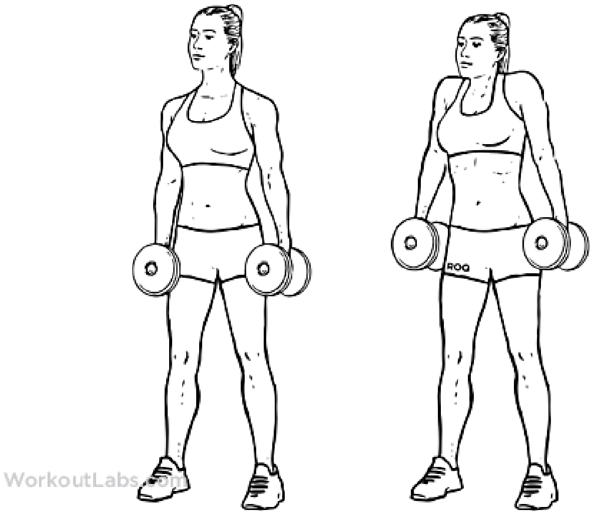

ALTERNATIVE: Shoulders to Ears

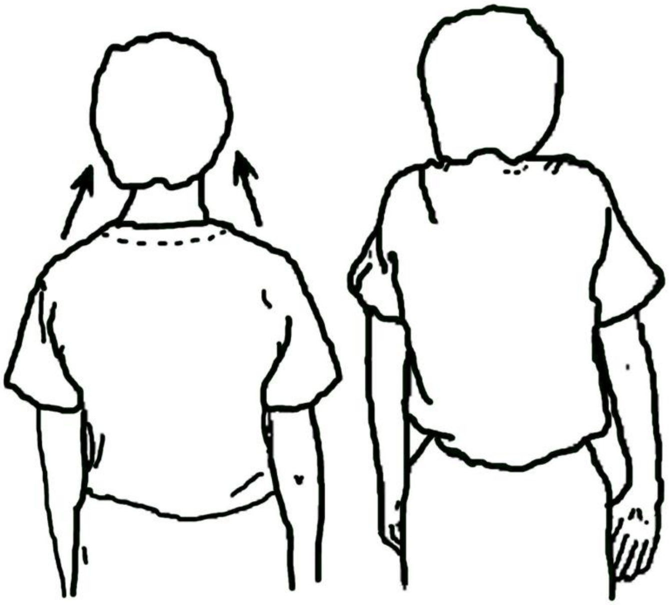

Goblet Squats with Dumbbell:

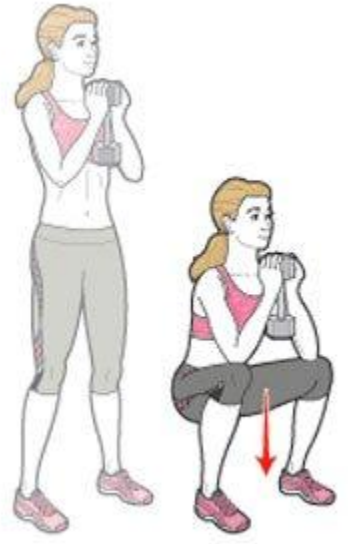

ALTERNATIVE: Air Squats

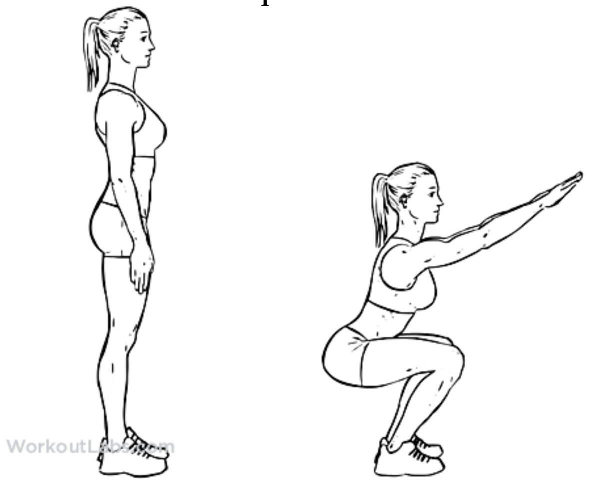

Hamstring Curls:

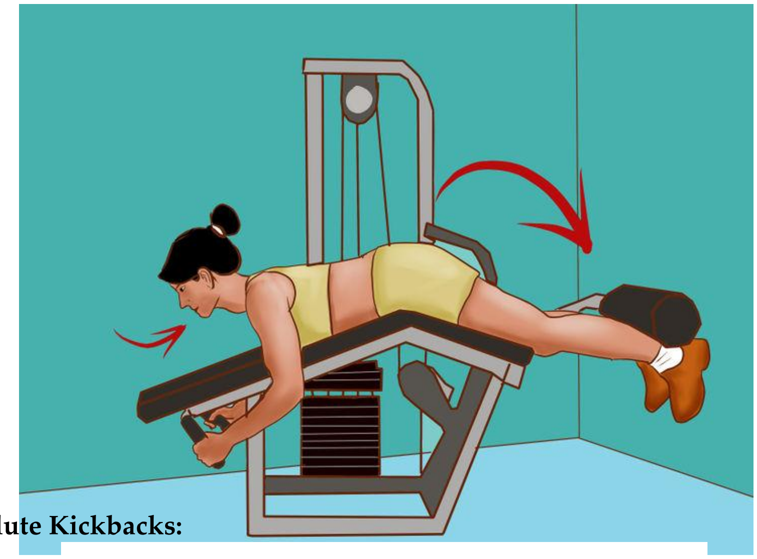

ALTERNATIVE: Glute Kickbacks:

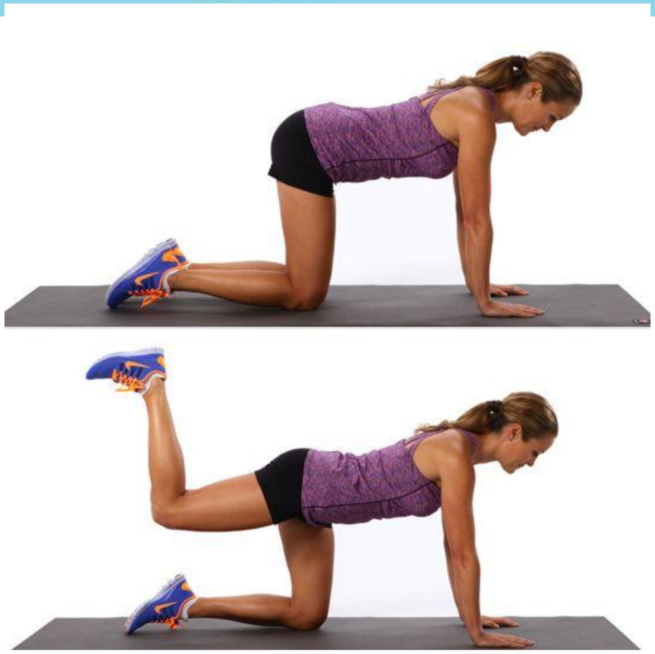

Jack Knives:

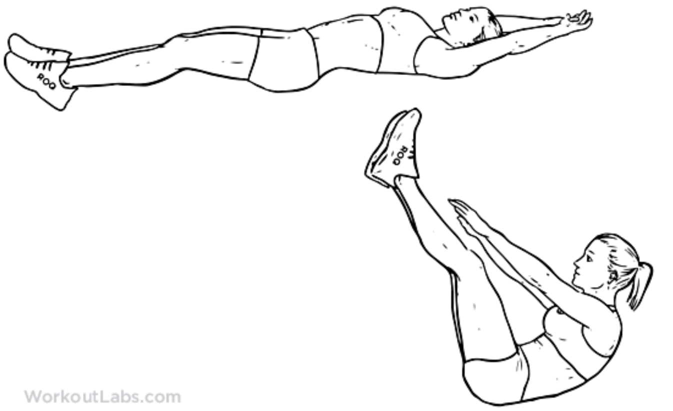

ALTERNATIVE: Standard Crunch

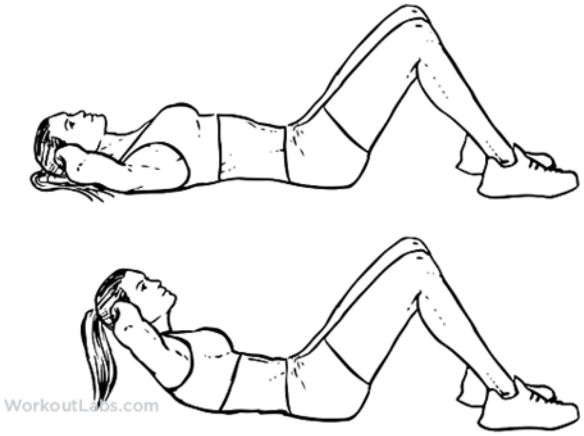

Lat Pulldowns:

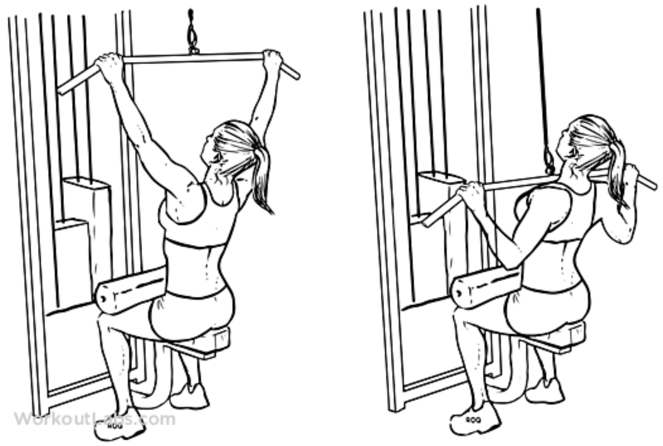

ALTERNATIVE: Seated Reaching Exercise

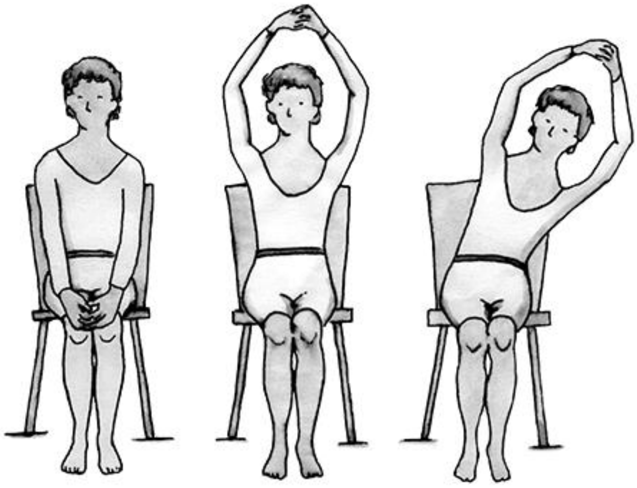

Leg Raises:

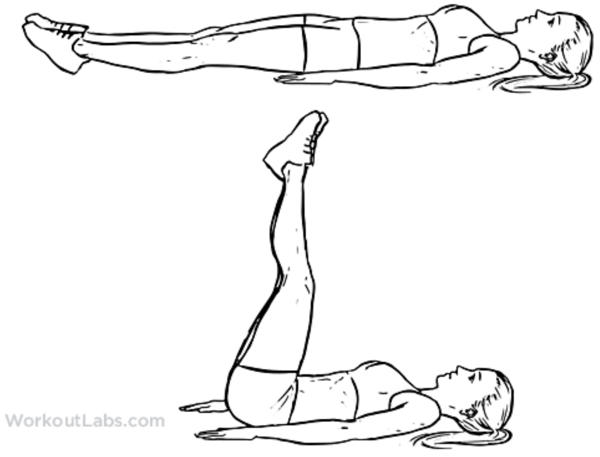

ALTERNATIVE: Seated Leg Lifts

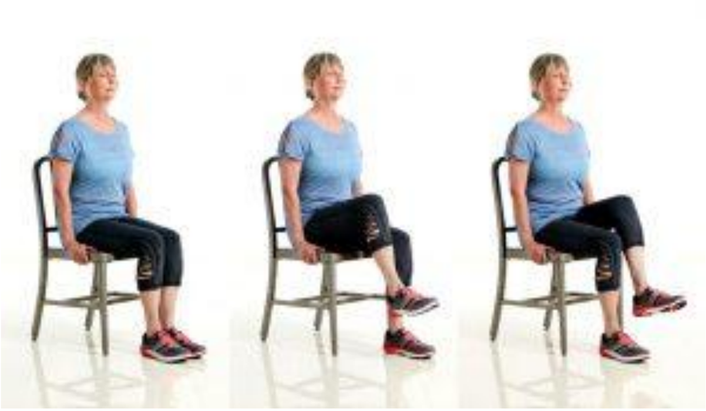

One-Arm Rows:

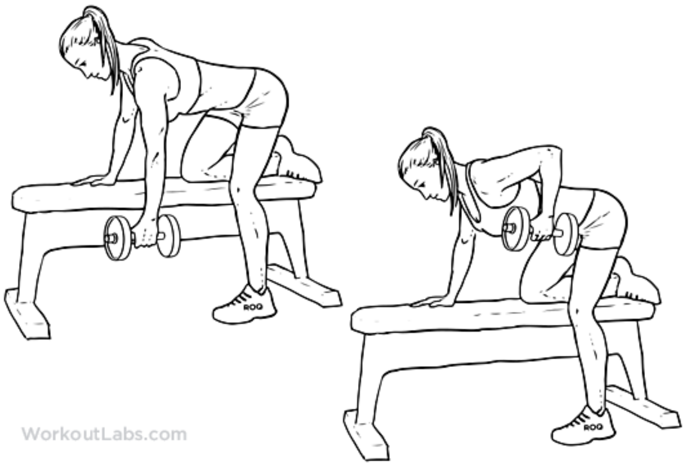

ALTERNATIVE: Single Arm Circles:

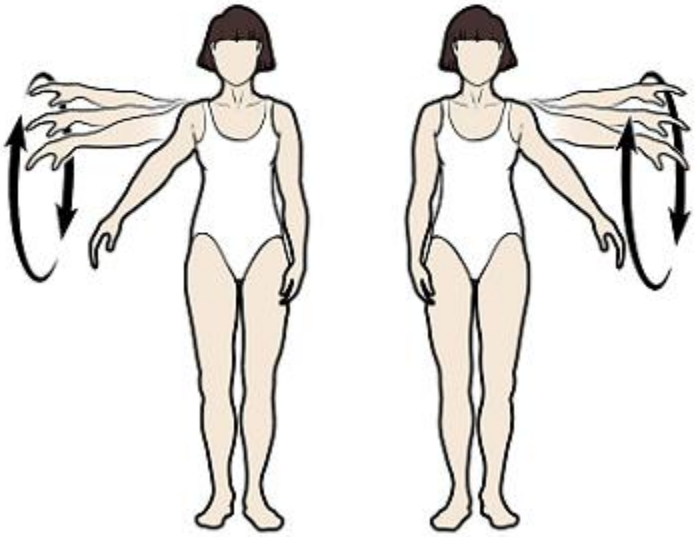

Seated Cable Row:

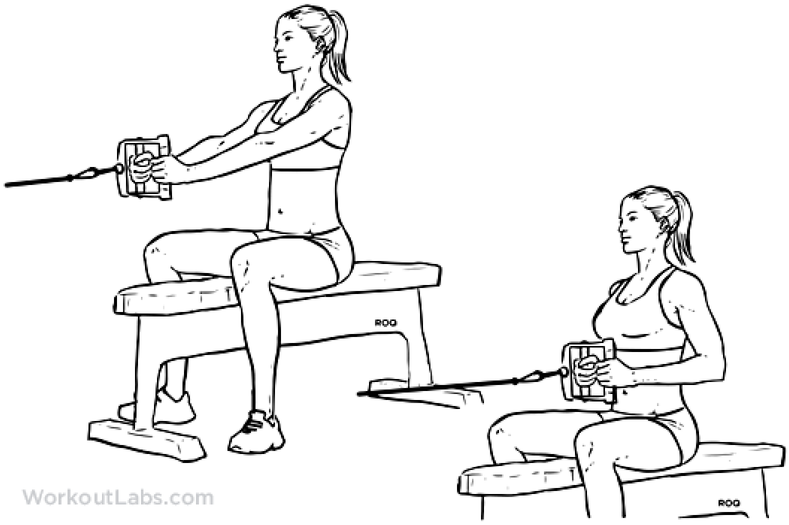

ALTERNATIVE: Single Arm Circles

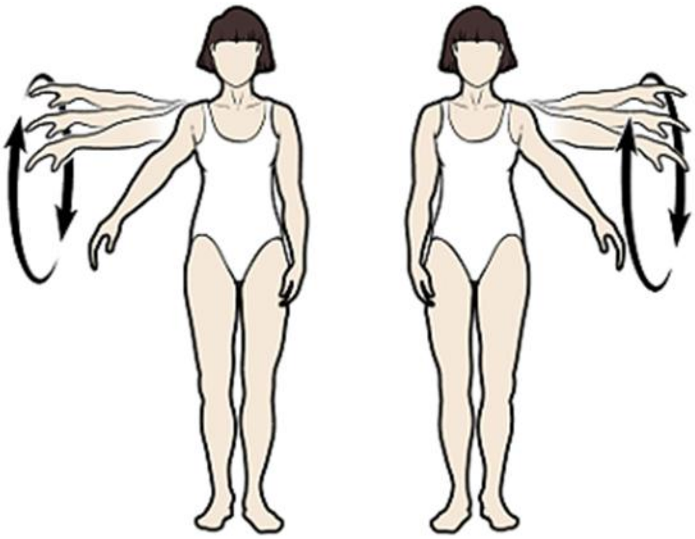

Sit-Ups:

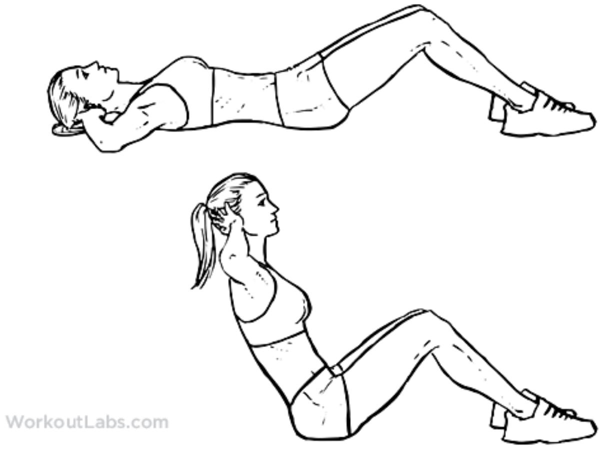

ALTERNATIVE: Standard Crunch:

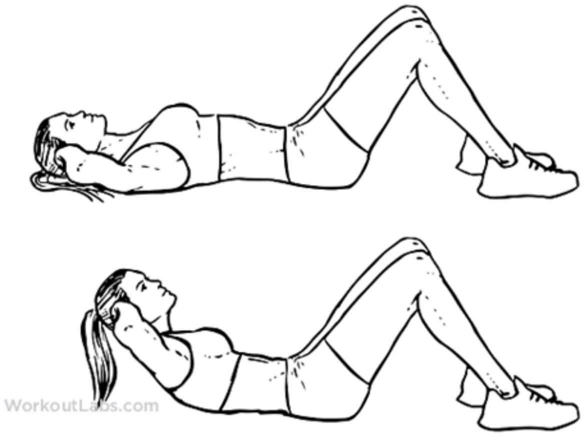

Tricep Kickbacks:

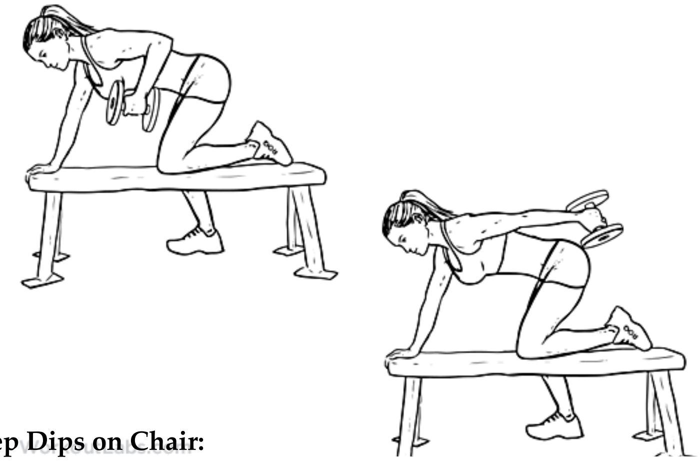

ALTERNATIVE: Tricep Dips on Chair:

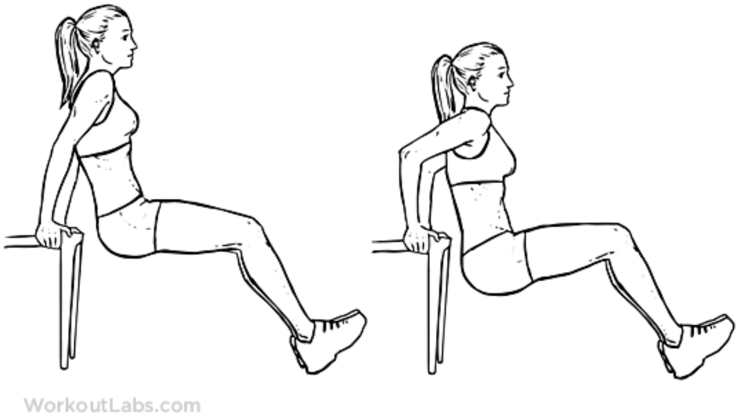

Upright Row:

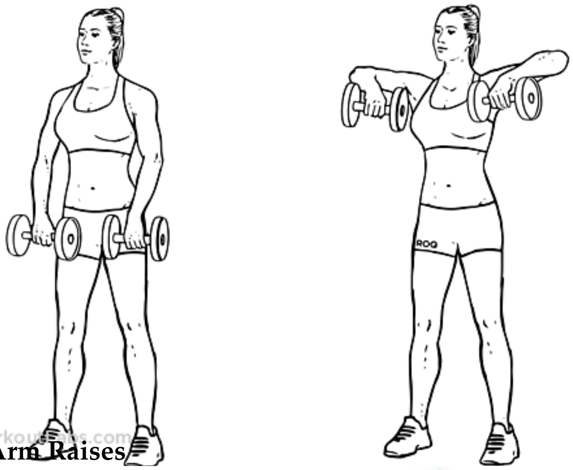

ALTERNATIVE: Forward Arm Raises

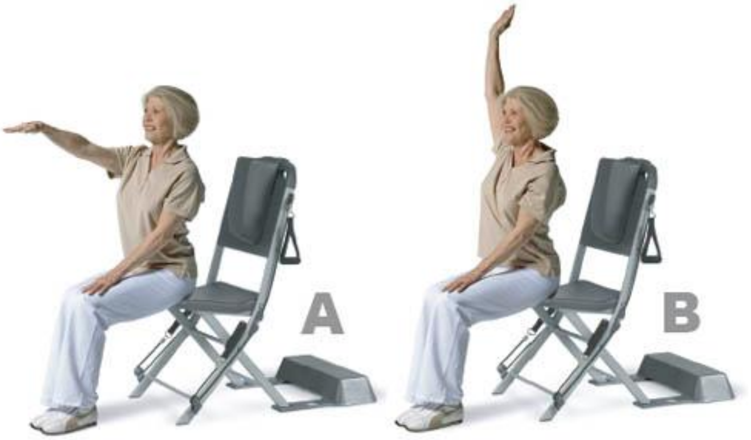

Supplement: Supplementary file 1 [file jcm-07-00140-s001.pdf]
